# Inorganic Phosphate Is Associated with RB1–E2F-Related Transcriptional and DNA Repair-Associated Changes Under Cisplatin Exposure in MDA-MB-231 Cells

**DOI:** 10.3390/cimb48080839

**Published:** 2026-08-18

**Authors:** Xiao Yang, Lihong Zhang, Xueqian Li, Hongfei Song, Fengmin Zhang, Lei Jiang, Wuqi Song

**Affiliations:** 1 Heilongjiang Provincial Key Laboratory of Infection and Immunity, Department of Microbiology, School of Basic Medical Sciences, Harbin Medical University, 157 Baojian Road, Nangang District, Harbin 150081, China; 13980718594@163.com (X.Y.);; 2 Department of Basic Medical Sciences, School of Basic Medical Sciences, Harbin Medical University, Harbin 150081, China; 3 Department of Pathology, The Second Affiliated Hospital of Harbin Medical University, Harbin 150086, China

**Keywords:** inorganic phosphate, cisplatin, RB1, E2F signaling, DNA repair, triple-negative breast cancer

## Abstract

Triple-negative breast cancer (TNBC) lacks effective targeted therapeutic strategies, and cisplatin resistance remains a major challenge in clinical treatment. This study aimed to investigate whether inorganic phosphate (Pi) influences cellular responses to cisplatin and to explore the potential molecular mechanisms involving RB1–E2F signaling, DNA repair regulation, and oxidative stress. Clinical associations between serum biochemical parameters and tumor histologic grade were evaluated in 246 patients with invasive ductal carcinoma. Triple-negative breast cancer cell line (MDA-MB-231), estrogen receptor-positive breast cancer cell line (MCF-7), and non-tumorigenic breast epithelial cell line (MCF-10A) were treated with control, Pi, cisplatin, or Pi combined with cisplatin.. Cellular proliferation, cell-cycle distribution, DNA damage, intracellular reactive oxygen species (ROS), inflammatory responses, and transcriptomic alterations were assessed using functional assays and RNA sequencing-based analyses. Pi levels showed an inverse association with tumor grade. In MDA-MB-231 cells, Pi combined with cisplatin resulted in enhanced growth inhibition, increased DNA damage accumulation, elevated cellular ROS production, and activation of inflammatory responses compared with cisplatin alone. Transcriptomic analyses revealed alterations in RB1–E2F-related transcriptional programs and reduced expression of DNA repair-associated gene sets. TCGA-BRCA analysis further indicated that elevated DNA repair pathway activity was associated with unfavorable survival outcomes. These findings suggest that Pi may modulate cisplatin responses through coordinated regulation of RB1–E2F signaling, DNA repair capacity, and oxidative stress responses, providing a potential mechanistic basis for further investigation of phosphate-associated therapeutic strategies in TNBC.

## 1. Introduction

Triple-negative breast cancer (TNBC) is an aggressive subtype of breast cancer characterized by the absence of estrogen receptor (ER), progesterone receptor (PR), and human epidermal growth factor receptor 2 (HER2) expression. Accounting for approximately 10–15% of all breast cancer cases, TNBC is associated with high histological grade, early metastasis, and poor clinical outcomes compared with other subtypes [1,2,3]. Epidemiological studies have also revealed marked disparities in TNBC incidence and prognosis across different populations, with higher prevalence and mortality observed in certain geographic regions and ethnic groups [4,5].

Due to the lack of established molecular targets, chemotherapy remains the cornerstone of TNBC treatment [6]. Among available agents, platinum-based drugs such as cisplatin have demonstrated significant clinical activity, particularly in patients with defects in DNA repair pathways, including BRCA1/2 alterations [7,8,9]. However, the clinical efficacy of cisplatin is often limited by intrinsic or acquired resistance, which reduces long-term therapeutic benefit and contributes to disease recurrence and progression [10,11].

Accumulating evidence suggests that the cellular response to cisplatin is closely linked to DNA damage response and repair mechanisms [12,13]. Tumor cells with enhanced DNA repair capacity can tolerate cisplatin-induced DNA lesions, thereby reducing drug sensitivity [14,15]. In addition, cell cycle regulation, particularly the RB1–E2F signaling axis, plays a critical role in coordinating DNA replication and DNA repair-related transcriptional programs [16,17,18]. However, the regulatory factors that modulate these processes in the context of cisplatin response remain incompletely understood.

Inorganic phosphate (Pi) is an essential cellular component involved in metabolic regulation, energy homeostasis, and intracellular signaling [19,20]. Emerging evidence suggests that elevated Pi levels can induce reactive oxygen species (ROS) production in cancer cells; however, its role in modulating chemotherapy response remains largely unexplored [21]. In this study, we investigated the association between Pi and cisplatin response in TNBC. We show that Pi treatment was associated with cellular changes under cisplatin exposure in MDA-MB-231 cells, accompanied by reduced RB1 phosphorylation, decreased RB1–E2F-related transcriptional activity, altered DNA repair-associated gene expression, and increased DNA damage accumulation. These findings support a model in which phosphate-related cellular responses may involve RB1–E2F-associated transcriptional programs and cisplatin-induced DNA damage.

## 2. Materials and Methods

### 2.1. Clinical Sample Collection and Grouping

This retrospective and non-invasive study was approved by the Medical Ethics Committee of the Second Affiliated Hospital of Harbin Medical University, and the requirement for informed consent was waived (Approval No. YJSKY2024-047). Clinical and pathological data were retrospectively collected from breast cancer patients treated at the Department of Breast Surgery, the Second Affiliated Hospital of Harbin Medical University, between 2022 and 2024. A total of 246 patients with invasive ductal carcinoma (IDC) who met the inclusion criteria were enrolled. All patients received standardized diagnosis and treatment and had complete laboratory blood test results and pathological information available. The inclusion criteria were pathologically confirmed IDC and complete clinical data. Exclusion criteria included traumatic fractures within the previous year, distant metastases to the brain or visceral organs, male breast cancer, coexistence of other malignancies, and incomplete clinical records. For subsequent statistical analyses, all clinical laboratory variables—including routine blood tests, urinalysis, liver and renal function indices, glucose, electrolytes, coagulation parameters, and carbohydrate antigens—were coded as categorical variables using SPSS version 26.0 (normal = 0; abnormal = 1). Based on pathological grading, patients were classified into three groups: grade I (*n* = 19), grade II (*n* = 111), and grade III (*n* = 116). In statistical analyses, Pearson correlation analysis was first performed to screen for preliminary associations among variables, with a significance threshold set at *p* < 0.05. Subsequently, logistic regression analysis was conducted to further evaluate differences among pathological grades, using *p* < 0.05 as the criterion for statistical significance.

### 2.2. Cell Culture and Drug Treatment

Human breast cancer cell lines MDA-MB-231 and MCF-7, and the normal mammary epithelial cell line MCF-10A, were obtained from Procell (Wuhan, China). Cells were cultured under standard conditions at 37 °C with 5% CO_2_ in appropriate media supplemented with 10% fetal bovine serum and antibiotics. Sodium phosphate (Na_2_HPO_4_/NaH_2_PO_4_) and cisplatin were dissolved in sterile water and diluted to the indicated concentrations prior to use. Cells were treated with inorganic phosphate (Pi, 5 mM), cisplatin (1 μM), or their combination. Cell passaging and treatment procedures were performed according to standard protocols.

### 2.3. Assessment of Cell Proliferation Using the iCELLigence Real-Time Cell Analysis System

For cell proliferation analysis, 150 μL of complete culture medium was added to each well of an 8-well E-plate L8 designed for cell proliferation assays. The plate was placed on the iCELLigence Real-Time Cell Analysis (RTCA) system (Agilent, Beijing, China) to ensure proper electrical contact. After confirming the experimental protocol and defining cell type and seeding density for each well in the system, baseline measurements were initiated. Once a stable baseline was achieved, cells were seeded at a density of 5 × 10^3^ cells per well. After stabilization, the E-plate L8 was removed, and 200 μL of cell suspension was added to each well. The plate was then returned to the incubator, and continuous monitoring of cell behavior was initiated. When cells reached a stable growth state, four experimental groups were established: control (NC), inorganic phosphate (Pi), cisplatin, and combined treatment (Pi + cisplatin). Cells were treated with the indicated concentrations (Pi group: 5 mM Pi; cisplatin group: 1 μM cisplatin; combination group: 5 mM Pi + 1 μM cisplatin), and real-time monitoring was continued. Cellular kinetic data were recorded at 1 h intervals. After cells entered the growth plateau phase, data were exported from the instrument and analyzed using iCELLigence Data Analysis Software version 1.0 to evaluate cell proliferation.

### 2.4. RNA Extraction and Real-Time Quantitative Polymerase Chain Reaction (RT–qPCR)

Total RNA was extracted using a commercial kit and reverse-transcribed into cDNA. Quantitative PCR was performed using SYBR-based detection. Relative gene expression levels were calculated using the 2^−ΔΔCt^ method with normalization to an internal reference gene. Primer sequences are listed in Supplementary Table S1.

### 2.5. Western Blot Analysis

Following the indicated treatments, cells were lysed using RIPA lysis buffer (Beyotime, Shanghai, China) supplemented with phenylmethylsulfonyl fluoride (PMSF; Meilun Biotechnology, Dalian, China) to inhibit protein degradation. Protein concentrations were determined using an enhanced bicinchoninic acid (BCA) protein assay kit (Beyotime, Shanghai, China). Equal amounts of protein were separated by SDS–PAGE and subsequently transferred onto polyvinylidene fluoride (PVDF) membranes. Membranes were blocked with TBST buffer containing 5% nonfat milk and then incubated overnight at 4 °C with primary antibodies. After washing with TBST, membranes were incubated with horseradish peroxidase (HRP)-conjugated secondary antibodies at room temperature. Protein signals were visualized using enhanced chemiluminescence (ECL) reagents and detected with a chemiluminescence imaging system. Band intensities were quantified by densitometric analysis using ImageJ software (ImageJ version 1.52; National Institutes of Health, NIH, Bethesda, MD, USA). The primary antibodies used in this study included β-Tubulin (Affinity, Changzhou, China), RB1 polyclonal antibody (Proteintech, Wuhan, China), phospho-RB1 (Ser807/811) polyclonal antibody (Proteintech, Wuhan, China), phospho-RB1 (Ser780) recombinant monoclonal antibody (Proteintech, Wuhan, China), RAD51 (Genuinbiotech, Hefei, China), and PCNA (Genuinbiotech, Hefei, China). β-Tubulin was used as an internal loading control for normalization.

### 2.6. Flow Cytometric Analysis of Reactive Oxygen Species (ROS) Levels and Cell Cycle Distribution

Following the indicated treatments, intracellular reactive oxygen species (ROS) levels and cell cycle distribution were analyzed by flow cytometry. Briefly, cells were washed with PBS and trypsin digestion was terminated using serum-free culture medium. After centrifugation, cell pellets were stained with either 2′,7′-dichlorodihydrofluorescein diacetate (DCFH-DA; 10 μmol/L) or propidium iodide (PI). For ROS measurement, cells were incubated with the DCFH-DA working solution for 20 min at 37 °C, with gentle mixing during incubation to ensure uniform probe–cell interaction. For cell cycle analysis, cells were fixed in 70% ethanol for 2 h, followed by staining with PI solution for 30 min. All samples were analyzed using a flow cytometer. Prior to acquisition, instrument voltage and compensation settings were adjusted using negative control samples to establish baseline signals. Data were subsequently analyzed using flow cytometry analysis software (FlowJo version 10.8.1).

### 2.7. RNA Sequencing and Gene Set Enrichment Analysis

MDA-MB-231 cells were treated with inorganic phosphate (Pi, 5 mM), cisplatin (1 μM), or their combination (Pi + cisplatin), while control cells received an equal volume of culture medium. Each group included four independent biological replicates. After 48 h of treatment, cells were harvested, and total RNA was extracted using TRIzol reagent (NCM Biotech, Suzhou, China). RNA concentration, purity, and integrity were assessed using an Agilent 2100 Bioanalyzer (Agilent, Beijing, China). Qualified RNA samples were submitted to Majorbio Bio-Pharm Technology Co., Ltd. (Shanghai, China) for library construction and transcriptome sequencing. Raw RNA-seq data were subjected to quality control and normalization using the Majorbio cloud-based analysis platform (Majorbio, Shanghai, China). Differentially expressed genes (DEGs) between each treatment group and the normal control (NC) group were identified using an adjusted *p* value < 0.05 and |log_2_ fold change| > 1 as the cutoff criteria. Visualization of DEGs and volcano plots was performed using the cloud analysis platform. Subsequently, Gene Ontology (GO) and Kyoto Encyclopedia of Genes and Genomes (KEGG) pathway enrichment analyses were conducted using the DAVID v6.8 database to identify the major biological processes and signaling pathways associated with the DEGs. Adjusted *p* values (Benjamini–Hochberg FDR) were used.

### 2.8. Comet Assay

To determine whether the Pi-associated attenuation of DNA repair-related responses is accompanied by altered DNA damage levels, a comet assay was performed in MDA-MB-231 cells following 48 h of treatment with NC, Pi, cisplatin, or Pi plus cisplatin. DNA damage was assessed using a standard alkaline comet assay following the manufacturer’s instructions with minor modifications. Briefly, cells were collected, washed with ice-cold PBS, and resuspended at a density of 1 × 10^6^ cells/mL. Slides were first coated with a layer of 1% normal melting point agarose and allowed to solidify. Subsequently, approximately 1 × 10^4^ cells were mixed with 0.7% low-melting-point agarose at 37 °C and spread onto the precoated slides to form the second layer. In some cases, a third agarose layer was added to improve gel stability. Slides were solidified at 4 °C. Cells were lysed in freshly prepared lysis buffer (Lysis Buffer: DMSO = 9:1) at 4 °C for 1–2 h. After lysis, slides were placed in alkaline electrophoresis buffer (1 mM EDTA, 200 mM NaOH, pH ~13) for 20–60 min to allow DNA unwinding. Electrophoresis was then performed at 25 V (approximately 0.75–1 V/cm) for 20–30 min under dark and cold conditions. Following electrophoresis, slides were neutralized with 0.4 M Tris-HCl (pH 7.5) and stained with propidium iodide for 10–20 min in the dark. After washing, slides were observed under a fluorescence microscope (Beyotime, Shanghai, China). Comet images were captured and analyzed to evaluate DNA damage.

### 2.9. Weighted Gene Co-Expression Network Analysis (WGCNA)

To elucidate the potential molecular regulatory networks induced by inorganic phosphate and its combination with cisplatin, weighted gene co-expression network analysis (WGCNA) was performed on the transcriptomic data. WGCNA was conducted using the WGCNA package (version ≥ 1.70) in R software (version ≥ 4.1.0). Prior to network construction, the expression matrix was preprocessed to remove genes with low expression levels and low variance across samples. Pairwise Pearson correlation coefficients were calculated to generate a correlation matrix, which was subsequently transformed into an adjacency matrix using an appropriate soft-thresholding power (β) to achieve scale-free network topology. A topological overlap matrix (TOM) was then constructed, followed by hierarchical clustering. Co-expression modules were identified using a dynamic tree-cutting algorithm. Module eigengenes (MEs) were calculated to represent the overall expression profiles of each module, and correlations between MEs and treatment groups were assessed to identify modules significantly associated with inorganic phosphate treatment for further analysis. Within the target modules, module membership (MM, also referred to as kME) and gene significance (GS) were calculated. Key module genes were subsequently selected for functional annotation and mechanistic investigation.

### 2.10. Gene Set Variation Analysis (GSVA)

Gene set variation analysis (GSVA) was performed to evaluate changes in pathway activity across different treatment groups (NC, Pi, cisplatin, and Pi + cisplatin). GSVA was conducted using the GSVA package in R software (version ≥ 4.1.0). Gene sets were obtained from the Kyoto Encyclopedia of Genes and Genomes (KEGG) database, with a particular focus on pathways related to metabolism, cell cycle regulation, and DNA repair. For each sample, enrichment scores (ESs) were calculated for the corresponding gene sets, and differences in pathway activity among treatment groups were visualized using heatmaps.

### 2.11. Gene Set Enrichment Analysis (GSEA)

To further validate pathway-level alterations and explore the effects of inorganic phosphate on MDA-MB-231 cells, gene set enrichment analysis (GSEA) was performed on the transcriptomic data. GSEA was conducted using the clusterProfiler package in R software. Gene sets were obtained from the Kyoto Encyclopedia of Genes and Genomes (KEGG) database, with a particular focus on pathways related to DNA repair, DNA replication, and cell cycle regulation. In addition, enrichment curves were generated for representative DNA repair pathways—including mismatch repair (MMR), base excision repair (BER), nucleotide excision repair (NER), and homologous recombination (HR)—across the comparisons of NC vs. Pi, NC vs. cisplatin, and cisplatin vs. Pi + cisplatin. These analyses were used to evaluate trends in pathway activity under different treatment conditions.

### 2.12. Construction of Protein–Protein Interaction (PPI) Networks

To further explore the functional associations among key module genes, protein–protein interaction (PPI) networks were constructed based on the STRING database. The resulting interaction networks were visualized using Cytoscape software (version ≥ 3.8.2). Network topology parameters were analyzed to identify potential hub gene clusters, which were subsequently used for functional annotation and mechanistic investigation.

### 2.13. Co-Expression Network Construction and Analysis of the RB1–E2F Regulatory Axis

To evaluate whether inorganic phosphate modulates DNA repair and cell cycle-related pathway activity through the RB1–E2F regulatory axis, an RB1–E2F functional module was constructed. This module included RB1; E2F family members (E2F1, E2F2, and E2F3); TFDP1 and TFDP2; as well as G1/S phase-related cyclin and cyclin-dependent kinase (CDK) genes (CCND1, CCND2, CCND3, CCNE1, CCNE2, CDK2, CDK4, and CDK6). Based on the normalized transcriptomic expression matrix, the average expression level of genes within the module was calculated as the RB1–E2F module activity score. Differences in module activity among treatment groups (NC, Pi, cisplatin, and Pi + cisplatin) were compared. Statistical analysis was performed using one-way analysis of variance (one-way ANOVA) to assess overall differences among the four groups, followed by pairwise comparisons when overall significance was observed. All statistical analyses were conducted in R software (version ≥ 4.1.0), and two-sided *p* values < 0.05 were considered statistically significant.

### 2.14. Gene-by-Gene Linear Regression Analysis

To assess the potential regulatory relationship between the RB1–E2F module and DNA repair pathways, a gene-by-gene linear regression analysis was performed. First, the RB1–E2F module activity score was calculated for each sample and used as the independent variable. Key DNA repair genes involved in four canonical pathways—mismatch repair (MMR), base excision repair (BER), nucleotide excision repair (NER), and homologous recombination (HR)—were curated from the KEGG database and included as dependent variables in the regression analysis.

For each repair gene, a linear regression model was constructed as follows:

Repair gene expression~RB1–E2F module score.

Linear regression analyses were performed using the lm() function in R software (version ≥ 4.1.0). Regression coefficients (β) were used to evaluate the direction and magnitude of the association between repair gene expression and RB1–E2F module activity, while *p* values were used to determine statistical significance. Based on the direction and significance of the β coefficients, repair genes were categorized as significantly positively correlated, significantly negatively correlated, or not significantly correlated, and the results were visualized accordingly.

### 2.15. TCGA Data Analysis

Transcriptomic data of breast cancer samples were obtained from the Genomic Data Commons (GDC) Data Portal (https://portal.gdc.cancer.gov/; accessed on 15 November 2024) and derived from The Cancer Genome Atlas (TCGA) project. The dataset included RNA-seq data from 1079 breast cancer tumor samples and 99 adjacent normal breast tissue samples. Breast cancer samples encompassed diverse clinical stages and molecular subtypes and were used to analyze transcriptomic differences between tumor tissues and matched normal tissues. Prior to downstream analyses, data were normalized using the DESeq2 package to reduce batch effects and technical noise, ensuring data quality and reliability. Subsequently, Gene Ontology (GO) and Kyoto Encyclopedia of Genes and Genomes (KEGG) pathway enrichment analyses were performed using the DAVID v6.8 database to identify the major biological processes and signaling pathways associated with differentially expressed genes.

### 2.16. Kaplan–Meier Overall Survival (OS) Analysis

To evaluate the prognostic impact of DNA repair pathway-related gene expression on breast cancer patient outcomes, expression-based scores for each DNA repair pathway were calculated for individual patients based on selected core gene sets. Patients were stratified into high-expression and low-expression groups according to the median score. Overall survival (OS) was analyzed using the Kaplan–Meier method, and survival differences between groups were assessed using the log-rank test. A *p* value < 0.05 was considered statistically significant. Survival curves were generated and visualized using the survival package in R software (version 4.1.0) to further investigate the prognostic value of different DNA repair pathways.

### 2.17. Statistical Analysis

All experimental data were analyzed using SPSS version 26.0 or R software version 4.1.0. Comparisons between two groups were performed using independent-samples t tests for normally distributed data or the Mann–Whitney U test for non-normally distributed data. Comparisons among three or more groups were conducted using one-way analysis of variance (ANOVA). When ANOVA indicated statistical significance, post hoc pairwise comparisons were performed using Tukey’s honestly significant difference (HSD) test. Paired or repeated-measures data were analyzed using paired t tests or repeated-measures ANOVA, as appropriate. Correlations between variables were assessed using Pearson correlation analysis or Spearman rank correlation analysis, depending on data distribution. Linear regression and logistic regression analyses were applied to evaluate relationships between variables across different treatment groups. All statistical tests were two-sided, and *p* values < 0.05 were considered statistically significant (* *p* < 0.05; ** *p* < 0.01; *** *p* < 0.001; **** *p* < 0.0001).

## 3. Results

### 3.1. Clinical Association Between Serum Phosphate and Pathological Grade

A total of 246 patients with invasive ductal carcinoma (IDC) of the breast were included in this study. All cases were diagnosed and treated at the Second Affiliated Hospital of Harbin Medical University between 2022 and 2024 (Approval No. YJSKY2024-047). Through retrospective analysis, general demographic information and comprehensive clinical laboratory data were systematically collected, including routine blood tests, liver and renal function parameters, glucose levels, electrolyte profiles, coagulation indices, and carbohydrate antigen levels.

**Figure 1:**
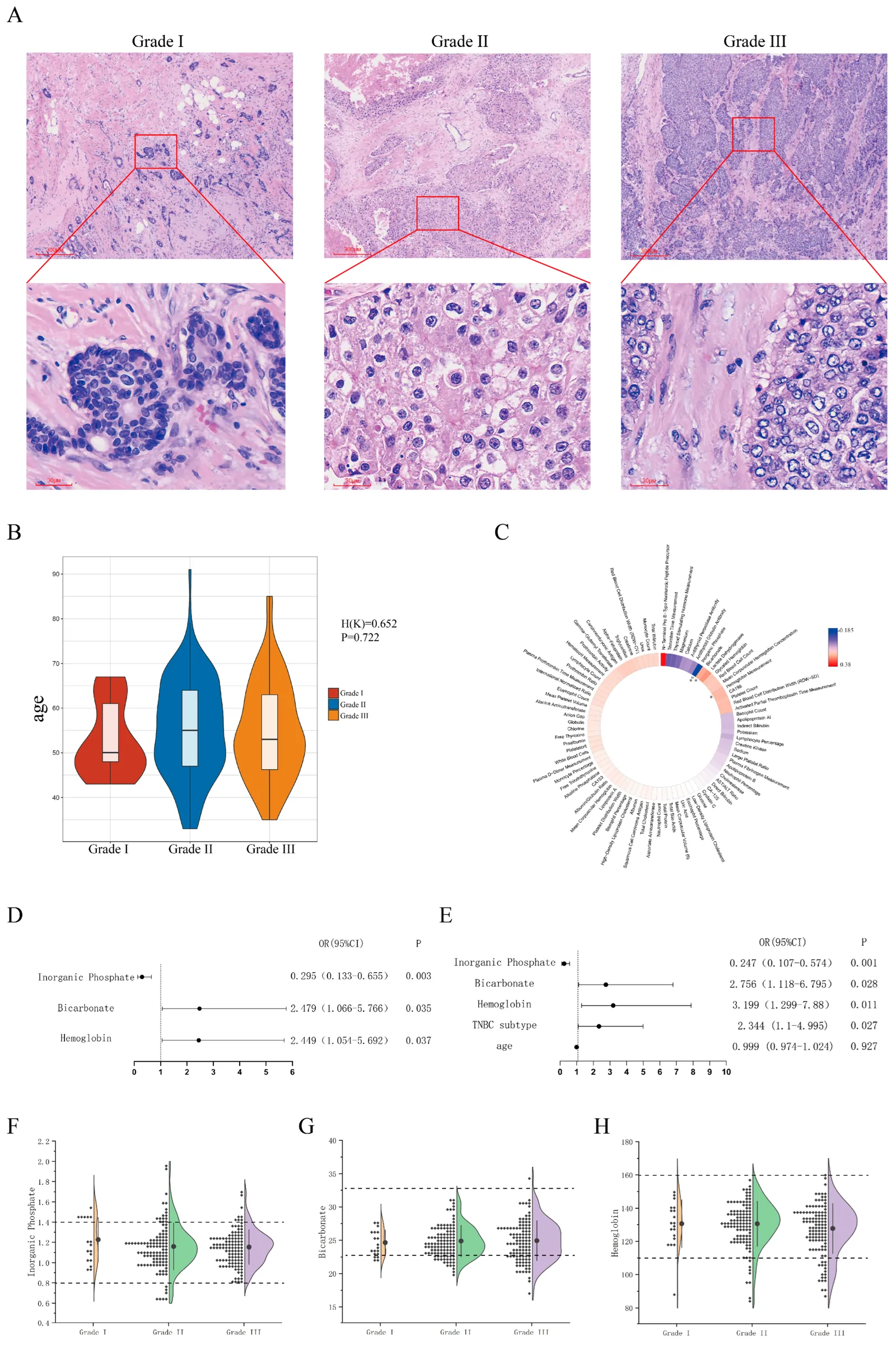
Association between Nottingham histological grade and laboratory indicators in breast invasive ductal carcinoma. (**A**) Representative hematoxylin and eosin (H&E)-stained sections of breast invasive ductal carcinoma illustrating the morphological features used for Nottingham histological grading. Upper panels show low-magnification views (40×) highlighting overall tumor architecture, while lower panels present high-magnification views (400×) depicting cytological characteristics, including glandular differentiation, nuclear pleomorphism, and mitotic activity, corresponding to Grade I, Grade II, and Grade III tumors. (**B**) Violin plot showing the age distribution of patients stratified by Nottingham histological grade (Grade I–III). (**C**) Circular heatmap displaying Pearson correlation coefficients between pathological grade and multiple laboratory indicators. Pathological grade was positively correlated with hemoglobin and bicarbonate levels, whereas a significant negative correlation was observed with serum inorganic phosphate levels. (**D**) Forest plot summarizing univariate binary logistic regression analyses evaluating the associations between abnormal laboratory indicators (hemoglobin, bicarbonate, and inorganic phosphate) and high-grade tumors (Grade III). (**E**) Forest plot showing adjusted odds ratios and 95% confidence intervals derived from multivariable binary logistic regression analysis of factors associated with high-grade tumors. The model included abnormal hemoglobin status, abnormal bicarbonate status, abnormal inorganic phosphate status, TNBC status, and age. Abnormal hemoglobin and bicarbonate status and TNBC subtype were positively associated with high-grade tumors, whereas abnormal inorganic phosphate status was inversely associated with high pathological grade. (**F**–**H**) Violin plots illustrating the distributions of inorganic phosphate (**F**), bicarbonate (**G**), and hemoglobin (**H**) levels across different Nottingham histological grades (Grade I–III). Significant differences were observed among grades, suggesting their potential association with pathological characteristics. Data are presented as mean ± SD. Statistical analyses were performed using ordinary one-way ANOVA or non-parametric tests where appropriate, followed by post hoc multiple-comparison correction. Statistical significance is indicated as * *p* < 0.05, ** *p* < 0.01.

Patients were graded according to the Nottingham histological grading system based on tumor morphological features and were classified into three groups: grade I (*n* = 19), grade II (*n* = 111), and grade III (*n* = 116) (Figure 1A). No significant difference in age was observed among patients with different histological grades (H = 0.652, *p* = 0.722) (Figure 1B).

Pearson correlation analysis was performed to explore associations between pathological grade and laboratory parameters. The results showed that pathological grade was positively correlated with hemoglobin levels (r = 0.131, *p* < 0.05) and bicarbonate levels (r = 0.154, *p* < 0.05), and negatively correlated with inorganic phosphate levels (r = −0.214, *p* < 0.05) (Figure 1C).

For categorical analyses, laboratory parameters were coded as normal = 0 and abnormal = 1, with abnormal values defined as measurements outside the reference range (including both below and above normal limits). These results indicate that pathological grade is associated with variations in multiple clinical laboratory parameters.

Based on the Nottingham grading system, patients were further stratified into low-grade tumors (grades I–II, *n* = 130) and high-grade tumors (grade III, *n* = 116). Tumor grade was treated as the dependent variable (high grade = 1, low grade = 0), while abnormalities in hemoglobin, bicarbonate, and inorganic phosphate levels were included as independent variables.

Univariate binary logistic regression analysis showed that abnormalities in hemoglobin and bicarbonate were associated with odds ratios (ORs) greater than 1, whereas abnormal inorganic phosphate status showed an OR < 1 (Figure 1D).

Considering the molecular heterogeneity of breast cancer, we further analyzed the distribution of abnormal phosphate status across different molecular subtypes. The proportion of patients with abnormal phosphate status was 16.0% in Luminal A, 10.0% in Luminal B, 11.4% in HER2-positive tumors, and 30.0% in TNBC (Figure S1A). Numerically, the TNBC subgroup exhibited the highest proportion of abnormal phosphate status among the molecular subtypes examined.

We next evaluated the association between phosphate status and pathological grade within the TNBC subgroup. The proportion of patients with abnormal phosphate status decreased across increasing histological grades, from 60.0% in grade I to 42.9% in grade II and 16.7% in grade III tumors, suggesting an inverse trend between phosphate abnormality and pathological grade (Figure S1B). Binary logistic regression restricted to TNBC cases further showed that abnormal phosphate status was inversely associated with high pathological grade (OR = 0.211, 95% CI: 0.051–0.871, *p* = 0.031) (Figure S1C).

To account for the potential influence of molecular subtype on pathological grade, age and TNBC status were subsequently included as clinically relevant covariates in the multivariable logistic regression model. After adjustment, abnormal hemoglobin status remained positively associated with high pathological grade (OR = 3.199, 95% CI: 1.299–7.880, *p* = 0.011), as did abnormal bicarbonate status (OR = 2.756, 95% CI: 1.118–6.795, *p* = 0.028). In contrast, abnormal inorganic phosphate status remained inversely associated with high pathological grade (OR = 0.247, 95% CI: 0.107–0.574, *p* = 0.001). TNBC status was also independently associated with high-grade tumors (OR = 2.344, 95% CI: 1.100–4.995, *p* = 0.027), whereas age was not significantly associated with pathological grade (OR = 0.999, 95% CI: 0.974–1.024, *p* = 0.927) (Figure 1E).

Further comparisons of hemoglobin, bicarbonate, and inorganic phosphate levels across different pathological grades revealed significant differences among groups (Figure 1F–H). Inspection of phosphate distributions indicated that most abnormal phosphate measurements were attributable to elevated phosphate levels (Figure 1F).

Importantly, this cohort included IDC cases with heterogeneous molecular subtypes, and detailed treatment information was not incorporated into the current analysis. Therefore, these findings should be interpreted as associative rather than causal, and potential confounding factors cannot be excluded.

### 3.2. Pi Treatment Is Accompanied by Altered Antiproliferative Responses Under Low-Dose Cisplatin Exposure

Based on the comparatively higher prevalence of phosphate abnormality observed in TNBC patients in the clinical cohort, together with the independent association between TNBC status and high pathological grade, MDA-MB-231 cells were selected as the primary model for mechanistic investigation. MCF-7 breast cancer cells and non-tumorigenic MCF-10A mammary epithelial cells were included as comparative cellular contexts in the real-time proliferation assay. Cells were divided into four groups: control (NC), inorganic phosphate treatment (Pi, 5 mM), cisplatin treatment, and combination treatment (Pi + cisplatin). Approximately 5 × 10^3^ cells were seeded per well in E-Plate L8 plates, and cell index (CI) values were recorded continuously after treatment until reaching the plateau phase. To quantitatively compare proliferation differences among groups, the area under the curve (AUC) of the real-time growth curves was calculated.

**Figure 2:**
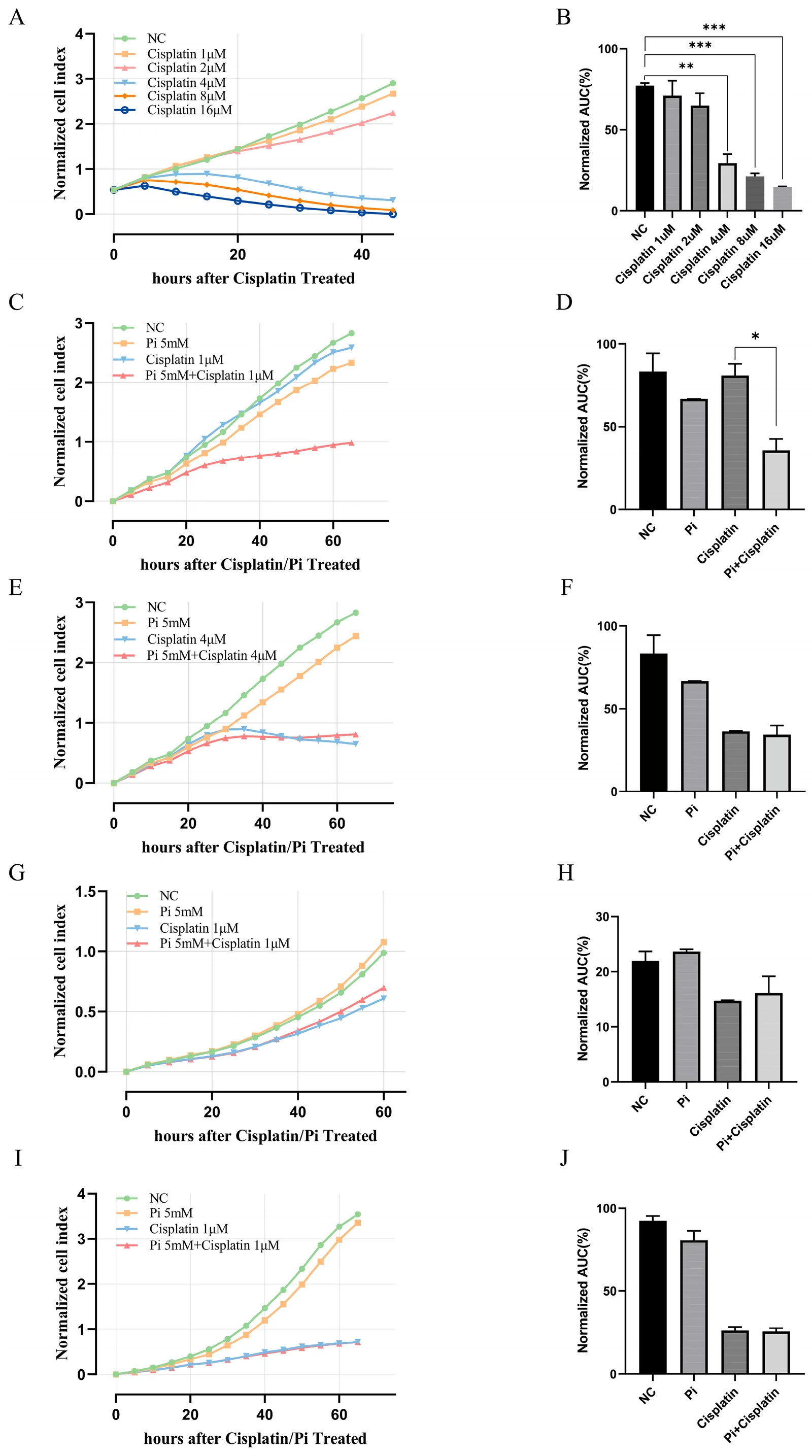
Real-time monitoring of cell proliferation using the iCELLigence system. (**A**) Dose-dependent proliferation curves of MDA-MB-231 cells treated with increasing concentrations of cisplatin. (**B**) Quantitative analysis of MDA-MB-231 cell proliferation based on area under the curve (AUC) values. (**C**) Proliferation curves of MDA-MB-231 cells under NC, Pi (5 mM), cisplatin (1 μM), and combined Pi (5 mM) + cisplatin (1 μM) treatment conditions. (**D**) Quantitative AUC analysis of MDA-MB-231 cells under the indicated low-dose cisplatin treatment conditions. (**E**) Proliferation curves of MDA-MB-231 cells treated with cisplatin (4 μM) alone or in combination with Pi (5 mM). (**F**) Quantitative AUC analysis of MDA-MB-231 cells under cisplatin (4 μM) alone or combined with Pi (5 mM). (**G**) Proliferation curves of MCF-7 cells under different treatment conditions. (**H**) Quantitative AUC analysis of MCF-7 cells. (**I**) Proliferation curves of MCF-10A cells under different treatment conditions. (**J**) Quantitative AUC analysis of MCF-10A cells. AUC values were calculated from real-time cell index (CI) curves and analyzed using the Mann–Whitney U test. * *p* < 0.05; ** *p* < 0.01; *** *p* < 0.001.

In MDA-MB-231 cells, dose–response analysis showed that cisplatin inhibited cell proliferation in a concentration-dependent manner, with a significant reduction in AUC observed at concentrations ≥ 4 μM compared with the control group (Figure 2A,B). In contrast, 1 μM cisplatin alone exerted only a modest inhibitory effect.

Under low-dose conditions, Pi (5 mM) alone resulted in a slight reduction in AUC, whereas 1 μM cisplatin alone did not show a clear inhibitory trend. Notably, the combination of Pi and 1 μM cisplatin led to a significantly greater reduction in AUC compared with cisplatin alone (Figure 2C,D). Furthermore, the AUC of the combination group was comparable to that observed with 4 μM cisplatin monotherapy, suggesting that Pi treatment may be associated with altered antiproliferative responses under low-dose cisplatin exposure in MDA-MB-231 cells.

**Figure 3:**
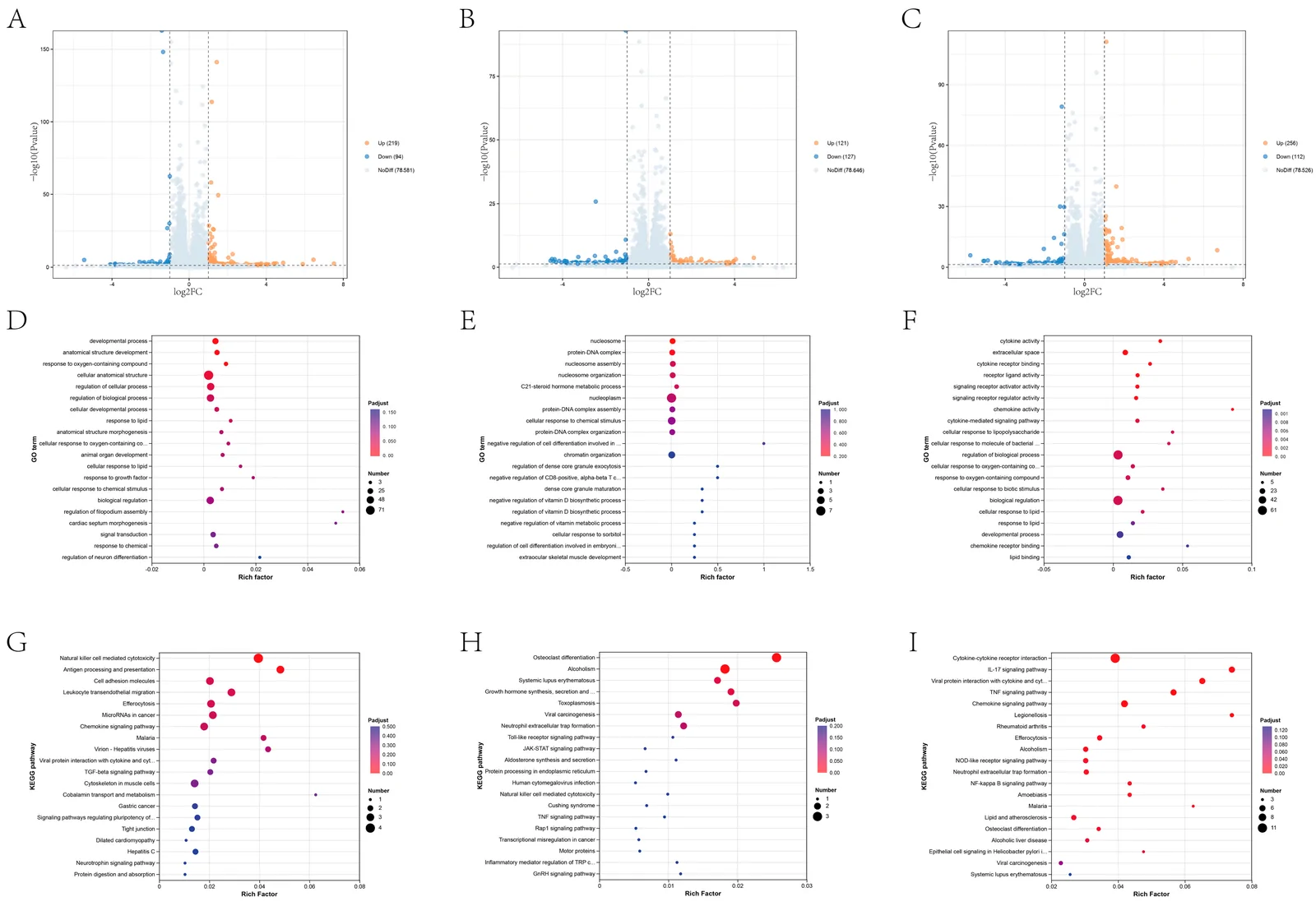
Transcriptomic profiling reveals treatment-associated inflammatory, stress-response, and immune-related transcriptional changes in MDA-MB-231 cells. (**A**–**C**) Volcano plots illustrating differentially expressed genes (DEGs) in MDA-MB-231 cells treated with inorganic phosphate (Pi, 5 mM) (**A**), cisplatin (1 μM) (**B**), or combined Pi and cisplatin treatment (**C**), each compared with the control group (NC). Differential expression analysis was performed using a cutoff of |log_2_ fold change| > 1 and *p* < 0.05. The horizontal dashed line indicates the significance threshold of *p* = 0.05 (−log10[*p*] ≈ 1.30), and the vertical dashed lines indicate the fold-change thresholds of log2FC = −1 and log2FC = 1. Red and blue dots represent significantly upregulated and downregulated genes, respectively, while gray dots indicate genes without significant changes. (**D**–**F**) Gene Ontology (GO) enrichment analyses of DEGs identified in MDA-MB-231 cells following Pi treatment (**D**), cisplatin treatment (**E**), and combined Pi plus cisplatin treatment (**F**), highlighting distinct biological processes and cellular functions associated with each condition. (**G**–**I**) Kyoto Encyclopedia of Genes and Genomes (KEGG) pathway enrichment analyses of DEGs from Pi-treated (**G**), cisplatin-treated (**H**), and Pi plus cisplatin–treated (**I**) MDA-MB-231 cells, revealing treatment-associated and potentially enhanced signaling pathways related to inflammation, immune response, oxidative stress, and cellular stress signaling.

To further assess whether Pi exerts an additional effect at higher cisplatin concentrations, cells were treated with 4 μM cisplatin alone or in combination with Pi. While 4 μM cisplatin alone markedly inhibited cell proliferation, the addition of Pi did not result in a further significant reduction in AUC (Figure 3E,F). These findings suggest that the Pi-associated effect appears more evident under low-dose cisplatin conditions, whereas no clear additive effect was observed when cisplatin alone already produced strong growth inhibition.

In contrast, in MCF-7 (Figure 2G,H) and MCF-10A cells (Figure 2I,J), cisplatin exhibited a general inhibitory trend, whereas Pi alone had minimal effects on AUC values. Moreover, the combination treatment did not produce a consistent additional reduction compared with cisplatin monotherapy. Among the three cellular contexts examined, the Pi-associated increase in the growth-inhibitory response under low-dose cisplatin exposure was most evident in MDA-MB-231 cells. However, because MCF-7 and MCF-10A do not represent independent TNBC models, these comparisons do not establish that the observed response is generalizable across TNBC. Accordingly, the subsequent mechanistic investigations should be interpreted as characterizing the response of the MDA-MB-231 model rather than representing a universal feature of TNBC, and validation in additional TNBC cell lines will be required in future studies.

### 3.3. Transcriptomic Reprogramming

To explore the potential molecular effects of inorganic phosphate (Pi) in combination with cisplatin, transcriptomic sequencing was performed in MDA-MB-231 cells. Differential gene expression analysis was conducted using a fold change > 2 and *p* < 0.05, and volcano plots were generated for each comparison. In MDA-MB-231 cells, compared with the control group (NC), Pi treatment resulted in 219 upregulated and 94 downregulated genes (Figure 3A); cisplatin treatment led to 121 upregulated and 127 downregulated genes (Figure 3B); and the combined Pi + cisplatin treatment yielded 258 upregulated and 112 downregulated genes (Figure 3C). Treatment groups containing Pi showed a higher number of differentially expressed genes, suggesting that Pi may be associated with broader transcriptional changes.

Gene Ontology (GO) enrichment analysis revealed that, in the NC vs. Pi comparison, enriched terms were mainly related to developmental and regulatory processes, including developmental process, anatomical structure development, and regulation of cellular processes, as well as responses to oxygen-containing compounds and lipids (Figure 3D). In the NC vs. cisplatin comparison, enriched GO terms were primarily associated with chromatin and nucleosome organization, including nucleosome, protein–DNA complex, chromatin organization, and nucleosome assembly, together with responses to chemical stimuli (Figure 3E). In the NC vs. Pi + cisplatin comparison, enriched GO terms included cytokine activity, chemokine activity, receptor–ligand activity, and cytokine-mediated signaling pathways, as well as extracellular region-related terms (Figure 3F). These enrichment patterns suggest that combined treatment is associated with transcriptional changes in inflammation-related and extracellular signaling processes.

KEGG pathway analysis showed that Pi treatment was associated with enrichment of pathways related to immune regulation and cell interaction, including natural killer cell–mediated cytotoxicity, antigen processing and presentation, leukocyte transendothelial migration, and chemokine signaling (Figure 3G). Cisplatin treatment was associated with enrichment of pathways related to chromatin function and stress responses, including systemic lupus erythematosus, viral carcinogenesis, and Toll-like receptor signaling (Figure 3H). Notably, the combined Pi and cisplatin treatment showed enrichment of multiple inflammation- and stress-related pathways, including cytokine–cytokine receptor interaction, IL-17 signaling, TNF signaling, chemokine signaling, NOD-like receptor signaling, and NF-κB signaling (Figure 3I). These findings indicate that the combined treatment is associated with broader transcriptional alterations involving stress- and inflammation-related gene sets.

### 3.4. WGCNA Identifies Pi-Associated Module

To further explore transcriptional patterns associated with inorganic phosphate (Pi), weighted gene co-expression network analysis (WGCNA) was performed using transcriptomic data from MDA-MB-231 cells. WGCNA constructs gene co-expression networks and groups genes into modules based on shared expression patterns, allowing identification of gene clusters associated with experimental conditions.

**Figure 4:**
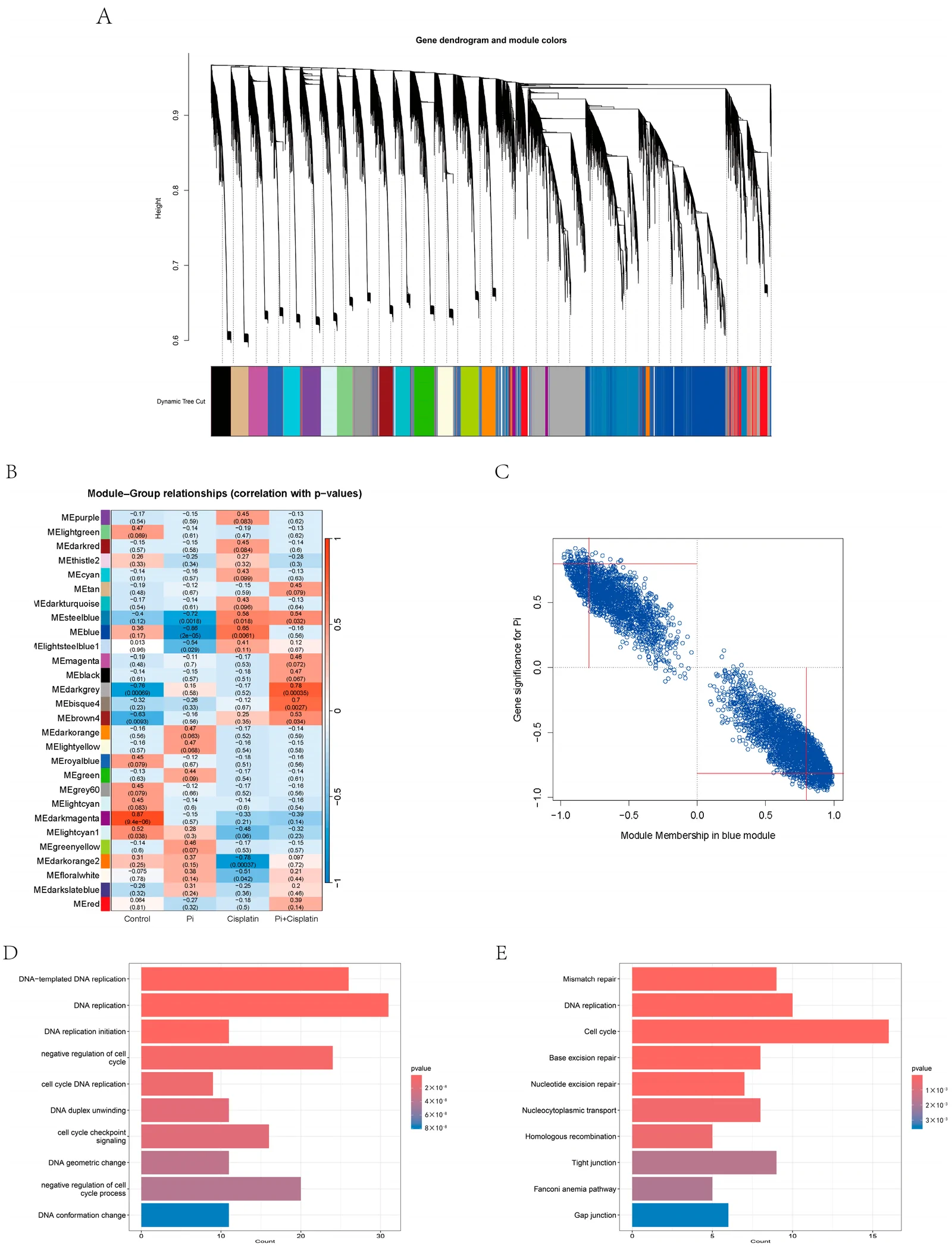
WGCNA reveals gene modules associated with inorganic phosphate treatment in MDA-MB-231 cells. (**A**,**B**) Weighted Gene Co-expression Network Analysis (WGCNA) was performed on transcriptomic data from MDA-MB-231 cells treated with inorganic phosphate (Pi) and cisplatin (Cis). A gene co-expression network was constructed, and 28 distinct modules were identified based on gene expression patterns using an appropriate soft threshold (β value). Each module represents a set of genes with similar expression trends, which may be involved in specific biological processes or pathways. The color scale represents the strength and direction of module–trait correlations, with red indicating positive correlations and blue indicating negative correlations. The intensity of the color reflects the correlation coefficient. (**C**) The MEblue module, which exhibited the highest correlation with Pi treatment, was identified as the most relevant module. Genes in this module were selected based on module membership (kME) and the absolute value of gene-trait significance (GS) greater than 0.8. Genes with high kME values are central to the module, and those with high GS values were found to have significant correlations with Pi treatment. (**D**) Gene Ontology (GO) enrichment analysis of genes in the MEblue module. (**E**) Kyoto Encyclopedia of Genes and Genomes (KEGG) pathway enrichment analysis of genes in the MEblue module.

A co-expression network was established, and an appropriate soft-thresholding power (β) was selected to achieve scale-free topology. A total of 28 modules were identified (Figure 4A,B). Among these, the MEblue module showed the strongest correlation with Pi treatment, suggesting that genes within this module may be associated with transcriptional responses to Pi. To further identify representative genes, those with module membership (kME) > 0.8 and gene–trait significance (GS) > 0.8 were selected, indicating strong connectivity within the module and strong association with Pi (Figure 4C).

Gene Ontology (GO) enrichment analysis of the selected genes revealed significant enrichment in biological processes related to DNA replication and cell cycle regulation, including DNA replication initiation, cell cycle, and DNA duplex unwinding (Figure 4D). These results indicate that Pi-associated gene modules are enriched for cell cycle- and DNA replication-related gene sets.

KEGG pathway analysis further showed enrichment in pathways related to DNA repair, including mismatch repair, base excision repair, nucleotide excision repair, and homologous recombination (Figure 4E). In addition, cell cycle-related pathways were also enriched.

Together, these findings suggest that Pi-associated transcriptional modules are enriched for gene sets involved in DNA replication, cell cycle regulation, and DNA repair-related processes. These observations provide a basis for subsequent analyses of DNA damage and repair-related responses under Pi and cisplatin treatment conditions.

### 3.5. GSVA/GSEA Indicates Reduced Enrichment of DNA Repair-Related Pathways

**Figure 5:**
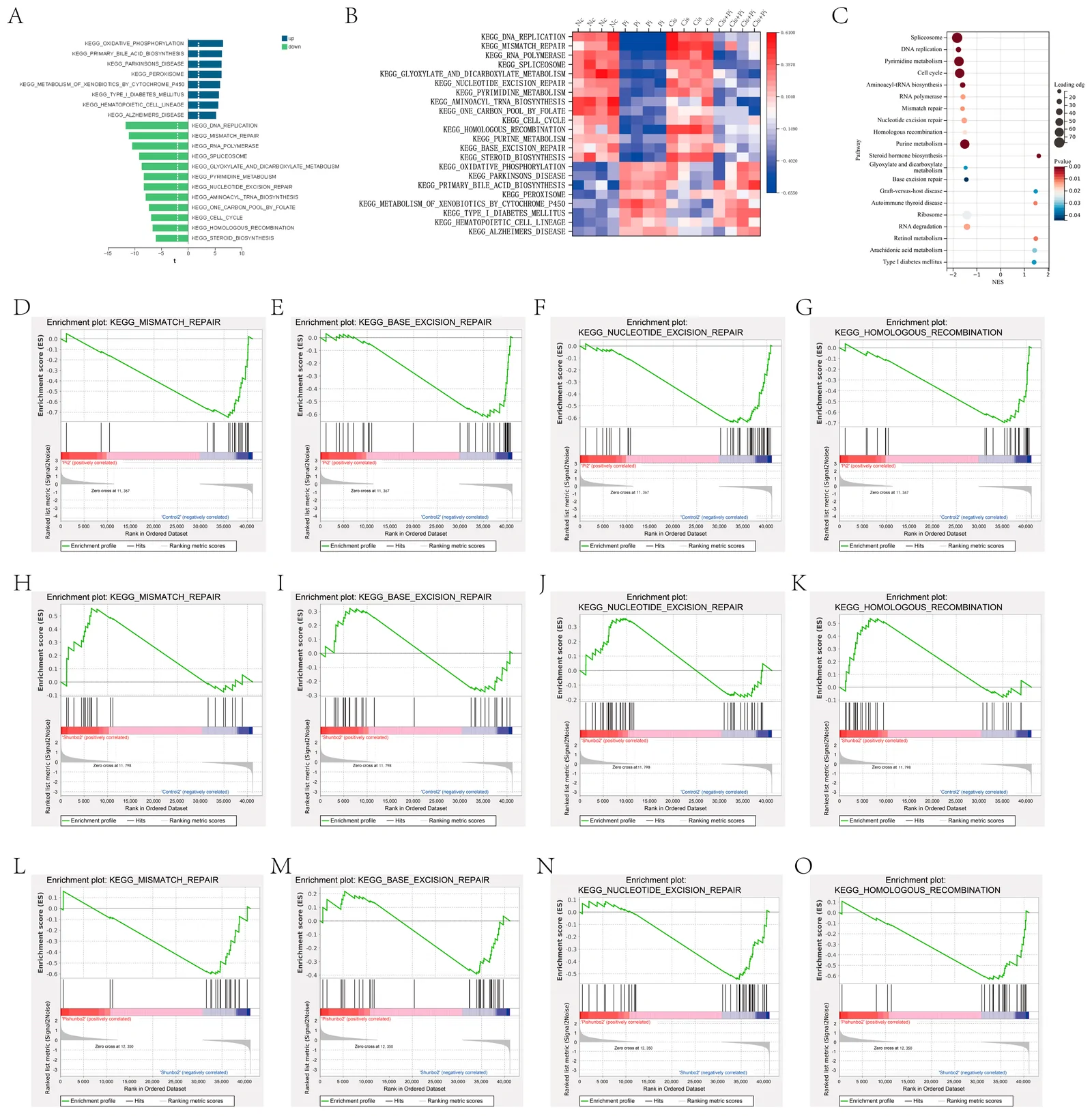
Inorganic phosphate is associated with reduced enrichment of DNA repair- and cell cycle-related pathways in MDA-MB-231 cells. (**A**) Gene Set Variation Analysis (GSVA)-based differential pathway activity analysis comparing the NC and Pi treatment groups, highlighting Pi-induced alterations in KEGG pathway enrichment scores. (**B**) Heatmap showing GSVA enrichment scores of representative KEGG pathways across four treatment groups (NC, Pi, cisplatin, and Pi plus cisplatin), illustrating distinct patterns of pathway activation related to oxidative metabolism, stress responses, DNA replication, and DNA repair. (**C**) Bubble plot summarizing Kyoto Encyclopedia of Genes and Genomes (KEGG) pathway enrichment results from Gene Set Enrichment Analysis (GSEA) comparing NC and Pi treatment groups, with significantly enriched pathways related to DNA repair, DNA replication, transcription, and cell cycle regulation highlighted. (**D**–**G**) GSEA plots showing enrichment of four DNA repair-related pathways—mismatch repair (**D**), base excision repair (**E**), nucleotide excision repair (**F**), and homologous recombination (**G**)—in the comparison between NC and Pi treatment groups. (**H**–**K**) GSEA plots of the same four DNA repair pathways—mismatch repair (**H**), base excision repair (**I**), nucleotide excision repair (**J**), and homologous recombination (**K**)—based on the comparison between NC and cisplatin treatment groups. (**L**–**O**) GSEA plots illustrating enrichment of mismatch repair (**L**), base excision repair (**M**), nucleotide excision repair (**N**), and homologous recombination (**O**) pathways in the comparison between cisplatin and Pi plus cisplatin treatment groups. In the enrichment plots, the red and blue colors in the phenotype bar indicate the two compared groups, with red representing genes associated with the first phenotype and blue representing genes associated with the second phenotype in the ranked list.

To further evaluate pathway-level changes associated with inorganic phosphate (Pi), GSVA-based KEGG pathway analysis was performed for the comparison of NC versus Pi (Figure 5A). The results indicated that Pi treatment was associated with alterations in multiple biological processes, including DNA repair, metabolism, cell cycle regulation, and immune-related pathways.

GSVA was further conducted across all four treatment groups (NC, Pi, cisplatin, and Pi + cisplatin), and representative pathway enrichment scores were visualized (Figure 5B). Pathways related to oxidative metabolism and stress responses showed relatively modest changes in the cisplatin-only group but exhibited a general upward trend in the Pi-treated and Pi + cisplatin groups.

In contrast, pathways involved in DNA replication and genomic maintenance—including DNA replication, mismatch repair, nucleotide excision repair, base excision repair, homologous recombination, and cell cycle—showed an overall upward trend in the cisplatin group, whereas a downward trend was observed in the Pi-treated group. Notably, in the Pi + cisplatin group, these pathways exhibited reduced enrichment compared with cisplatin alone.

To further validate these observations, KEGG-based GSEA was performed comparing the NC and Pi groups (Figure 5C). The enriched pathways were predominantly related to DNA replication, RNA processing, and cell cycle regulation, including spliceosome, DNA replication, mismatch repair, nucleotide excision repair, homologous recombination, and cell cycle.

Focused GSEA of four representative DNA repair pathways—mismatch repair (MMR), base excision repair (BER), nucleotide excision repair (NER), and homologous recombination (HR)—was subsequently performed. In the NC vs. Pi comparison (Figure 5D–G), all four pathways showed significant negative enrichment (NES < 0, *p* < 0.05), indicating reduced enrichment of DNA repair-related gene sets in the Pi-treated group. In the NC vs. cisplatin comparison (Figure 5H–K), these pathways showed a non-significant upward trend. In the cisplatin vs. Pi + cisplatin comparison (Figure 5L–O), negative enrichment was again observed across all four pathways.

Taken together, these findings suggest that Pi treatment is associated with decreased enrichment of multiple DNA repair-related gene sets, particularly in the context of cisplatin exposure. These observations may be consistent with altered DNA damage response-related patterns and provide a basis for subsequent functional analyses.

### 3.6. Pi Treatment Is Associated with G1-Phase Enrichment and Reduced RB1–E2F Module Scores

**Figure 6:**
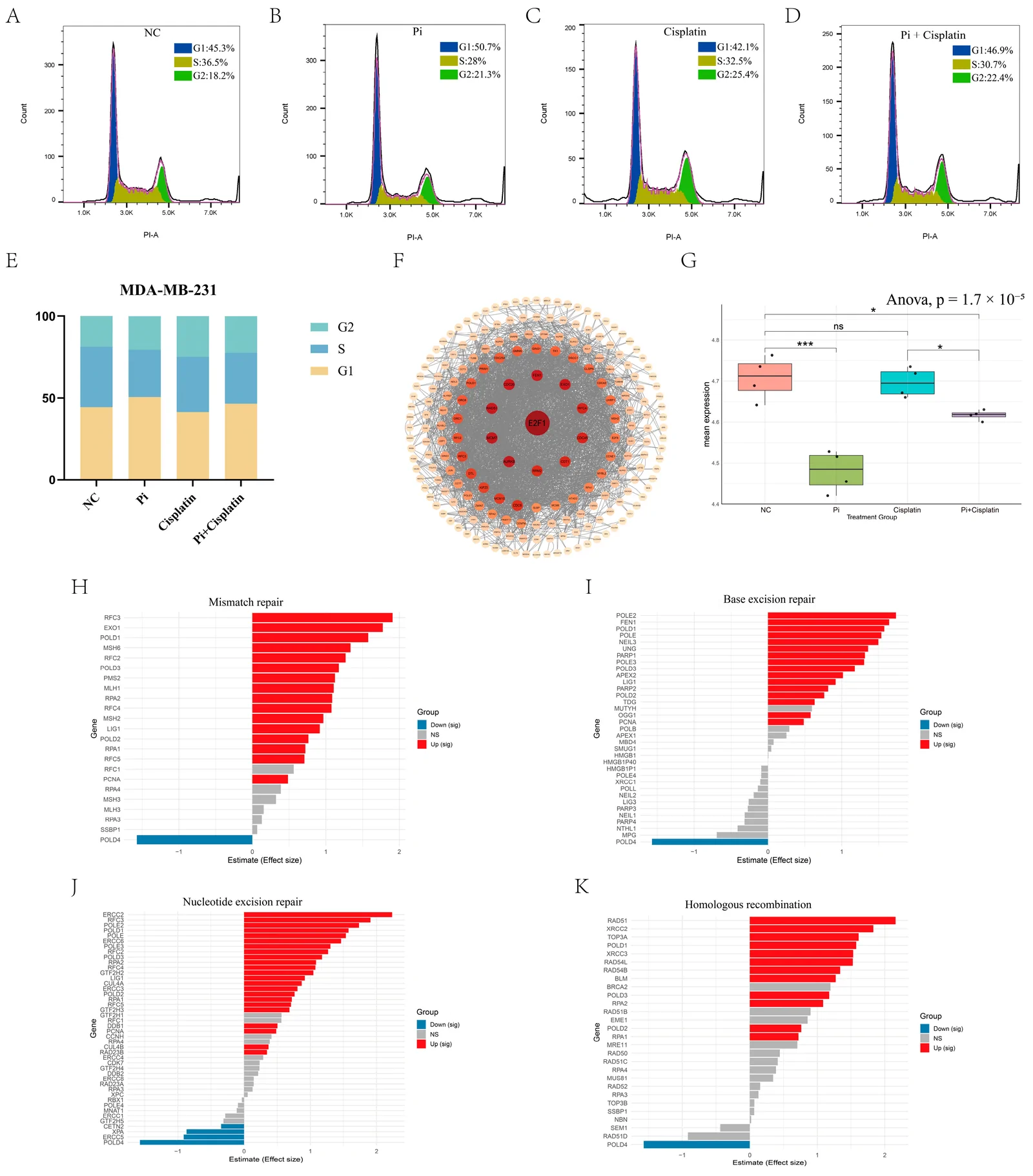
Inorganic phosphate induces G1-phase enrichment and is associated with reduced RB1–E2F module scores. (**A**–**D**) Cell-cycle distribution of MDA-MB-231 cells under negative control (NC), inorganic phosphate (Pi), cisplatin (Cisplatin), and combined Pi plus cisplatin (Pi + Cisplatin) treatments was analyzed by flow cytometry following propidium iodide (PI) staining. Representative DNA content histograms are shown. Different colors indicate the fitted G1, S, and G2/M cell-cycle phases, and the black line represents the fitted DNA content distribution generated by the FlowJo cell cycle analysis model. (**E**) Quantification of the percentages of cells in G1, S, and G2/M phases under each treatment condition. Data are summarized as stacked bar plots based on flow cytometry analysis. (**F**) Protein–protein interaction (PPI) network constructed from genes in the MEblue module. Nodes represent proteins and edges indicate predicted functional interactions; densely connected sub-networks reflect coordinated regulation of cell-cycle progression, DNA damage response, and DNA repair processes. (**G**) RB1–E2F module scores were calculated as the average expression score of RB1–E2F pathway genes (RB1, E2Fs, TFDPs, cyclins, and CDKs) in NC, Pi, Cisplatin, and Pi + Cisplatin groups. Statistical significance was assessed by one-way ANOVA. (**H**–**K**) Linear regression analyses examining the association between RB1–E2F module activity and the expression of core genes involved in four major DNA repair pathways: mismatch repair (MMR, (**H**)), base excision repair (BER, (**I**)), nucleotide excision repair (NER, (**J**)), and homologous recombination (HR, (**K**)). For each gene, the regression coefficient derived from the model gene expression~RB1–E2F module score is shown. Red indicates a significant positive association, blue indicates a significant negative association, and gray denotes non-significant relationships. Statistical significance is indicated as follows: ns, *p* > 0.05; * *p* < 0.05; *** *p* < 0.001.

To further evaluate the enrichment of pathways related to DNA replication and cell cycle regulation indicated by transcriptomic analyses, flow cytometry was performed to assess cell cycle distribution in MDA-MB-231 cells under different treatment conditions. Compared with the control group, cells treated with inorganic phosphate (Pi) exhibited an increased proportion of cells in the G1 phase, suggesting G1-phase arrest. In contrast, cisplatin-treated cells showed accumulation in the G2 phase, consistent with activation of the G2 checkpoint following DNA damage. In the combined treatment group (Pi + cisplatin), the proportion of cells in the G1 phase increased compared with the cisplatin-only group, accompanied by a relative reduction in the G2-phase population. These results indicate that Pi is associated with a shift in cisplatin-induced cell cycle distribution toward G1-phase enrichment (Figure 6A–E).

Given the enrichment of pathways related to DNA replication, cell cycle regulation, and DNA repair in transcriptomic analyses, we next examined potential regulatory links between cell cycle control and DNA repair. The RB1–E2F signaling axis is a well-established regulator of G1/S-phase transition and controls the expression of genes involved in DNA replication and repair. Based on this framework and the observed G1-phase accumulation, we hypothesized that Pi-associated cell-cycle changes may involve RB1–E2F-related transcriptional programs. However, E2F transcriptional activity was not directly assessed in this study. Accordingly, the observed reduction in RB1–E2F module scores should be interpreted as transcriptomic evidence of altered expression of an RB1–E2F-associated gene program rather than direct evidence of E2F1 transcriptional activity.

To further explore transcriptional organization, a protein–protein interaction (PPI) network was constructed using genes from the MEblue module. The network revealed enrichment of genes involved in cell cycle progression, DNA damage response, and transcriptional regulation (Figure 6F). Although E2F1 was not the top-ranked hub gene by network topology, it occupied a central position based on its known biological role as a regulator of DNA replication and repair-related genes. These observations suggest that the Pi-associated gene module may be enriched for gene sets linked to an E2F-centered regulatory network. An RB1–E2F-related gene module was constructed to represent an RB1–E2F-associated transcriptional program involved in G1/S-phase regulation, comprising RB1; E2F family members (E2F1, E2F2, and E2F3); dimerization partners (TFDP1 and TFDP2); and G1/S-phase-associated cyclins and cyclin-dependent kinases (CCND1, CCND2, CCND3, CCNE1, CCNE2, CDK2, CDK4, and CDK6). Comparison of RB1–E2F module scores across treatment groups revealed significant differences (one-way ANOVA, *p* = 1.7 × 10^−5^), with lower module scores observed in the Pi-treated group and a further decreasing trend in the combined treatment group (Figure 6G). This pattern suggests that Pi treatment is associated with reduced expression of an RB1–E2F-associated transcriptional program.

To assess the relationship between this module and DNA repair pathways, linear regression analyses were performed between RB1–E2F module scores and the expression of core DNA repair genes. Across multiple pathways—including mismatch repair, base excision repair, nucleotide excision repair, and homologous recombination—most genes showed positive associations with RB1–E2F module scores (β > 0, *p* < 0.05) (Figure 6H–K).

Taken together, these findings suggest that reduced expression of the RB1–E2F-associated transcriptional program is associated with decreased expression of DNA repair-related genes under Pi treatment. These observations are consistent with a potential link between cell cycle regulation and DNA repair modulation, although direct functional validation of RB1 phosphorylation status and E2F1 transcriptional activity (e.g., luciferase reporter assays) will be required to establish causality.

### 3.7. Pi Treatment Is Associated with Reduced RB1 Phosphorylation and Decreased PCNA/RAD51 and Putative E2F1-Related Repair Genes

**Figure 7:**
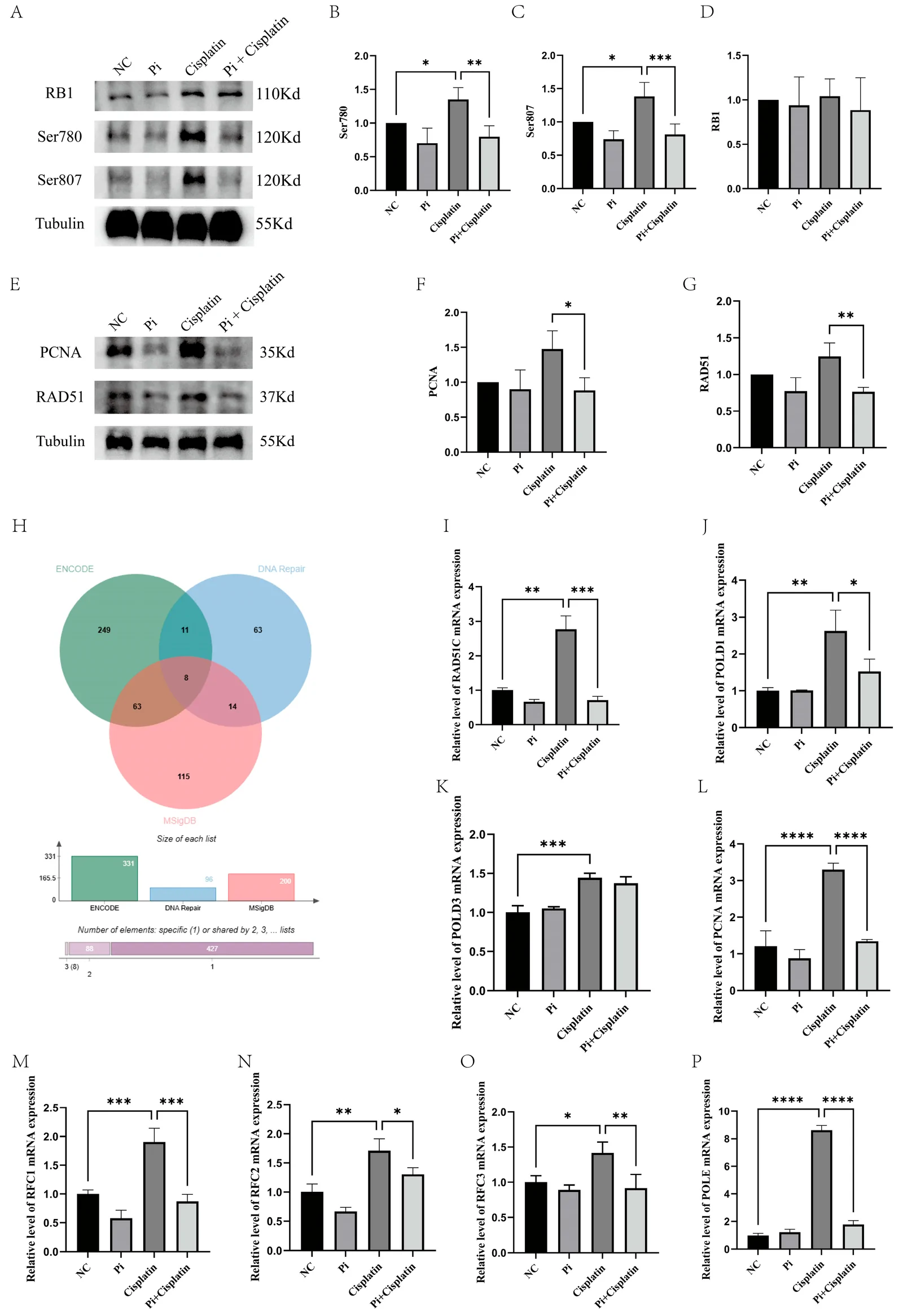
Inorganic phosphate is associated with reduced RB1 phosphorylation and decreased expression of putative E2F1-associated DNA repair genes under cisplatin treatment. (**A**) Representative Western blot images showing total RB1 and phosphorylated RB1 at Ser780 and Ser807 in MDA-MB-231 cells treated with vehicle control (NC), inorganic phosphate (Pi), cisplatin, or the combination of Pi and cisplatin for 48 h. Tubulin was used as a loading control. (**B**,**C**) Densitometric quantification of phosphorylated RB1 at Ser780 (**B**) and Ser807 (**C**), normalized to the corresponding loading control. (**D**) Densitometric quantification of total RB1 protein levels. (**E**) Relative protein expression levels of PCNA and RAD51 based on densitometric analysis of Western blot bands. (**F**,**G**) Quantitative analysis of PCNA (**F**) and RAD51 (**G**) protein expression levels in MDA-MB-231 cells under the indicated treatment conditions. (**H**) Identification of candidate E2F1-associated DNA repair genes by integrative analysis of publicly available E2F1 ChIP-seq datasets (ENCODE and ChIP-Atlas), canonical DNA repair pathways (MMR, BER, NER, and HR), and the MSigDB HALLMARK_E2F_TARGETS gene set. The overlap highlights genes with putative E2F1 binding and DNA repair-related functions. Different colors in the Venn diagram represent the three independent gene sets analyzed. The colored regions in the summary bar below indicate the distribution of genes according to their occurrence across datasets. From left to right, the regions represent genes shared by all three lists (n = 8), genes shared by two lists (n = 88), and genes specific to a single list (n = 427). (**I**–**P**) Quantitative real-time PCR (qPCR) analysis of mRNA expression levels of selected DNA repair-related genes (RAD51C, POLD1, POLD3, PCNA, RFC1, RFC2, RFC3, and POLE) in MDA-MB-231 cells treated with NC, Pi, cisplatin, or Pi plus cisplatin for 48 h. Data are presented as mean ± SD from at least three independent experiments. Statistical significance was determined using one-way ANOVA followed by multiple-comparison tests. * *p* < 0.05, ** *p* < 0.01, *** *p* < 0.001, **** *p* < 0.0001.

To further investigate the molecular changes associated with Pi treatment under cisplatin exposure, MDA-MB-231 cells were analyzed after 48 h of treatment. To determine whether Pi affects the functional state of retinoblastoma protein (RB1), phosphorylation of RB1 at Ser780 and Ser807 was assessed. Compared with cisplatin treatment alone, combined Pi and cisplatin treatment consistently reduced RB1 phosphorylation at both sites, whereas total RB1 protein levels did not show a consistent trend (Figure 7A–D). These results suggest that Pi is associated with reduced RB1 phosphorylation, consistent with a cellular state generally linked to increased RB1 activity.

Given that hypophosphorylated RB1 is generally associated with reduced E2F-dependent transcriptional programs, we next examined whether Pi influences downstream DNA repair-associated factors. Western blot analysis showed that the protein levels of PCNA and RAD51, two key components involved in DNA replication and homologous recombination repair, were reduced in the Pi plus cisplatin group compared with cisplatin treatment alone (Figure 7E–G). These findings provide protein-level evidence that Pi treatment was accompanied by reduced levels of selected DNA repair-associated proteins under cisplatin-induced DNA damage conditions.

To further explore potential E2F1-associated DNA repair genes, publicly available E2F1 ChIP-seq datasets from ENCODE and ChIP-Atlas were analyzed to identify candidate E2F1-bound targets. High-confidence genes (E2F1 |Average| ≥ 700) were intersected with genes annotated in four canonical DNA repair pathways—mismatch repair, base excision repair, nucleotide excision repair, and homologous recombination—as well as with the HALLMARK_E2F_TARGETS gene set from MSigDB. This integrative analysis yielded a subset of candidate genes, including PCNA, POLD3, RFC1, RFC2, RFC3, POLE, POLD1, and RAD51C, which represent putative E2F1-associated DNA repair-related genes inferred from public datasets (Figure 7H).

Based on this candidate gene set, mRNA expression levels were further examined by qPCR in the NC, Pi, cisplatin, and Pi plus cisplatin groups after 48 h of treatment (Figure 7I–P). Compared with cisplatin alone, combined Pi and cisplatin treatment significantly reduced the expression of RAD51C, POLD1, PCNA, RFC1, RFC2, RFC3, and POLE, whereas POLD3 expression did not show a significant difference. These findings indicate that Pi is associated with reduced expression of multiple DNA repair-related genes that are putative E2F1 targets.

These results are consistent with a model in which Pi-associated cellular changes under cisplatin exposure may involve reduced RB1 phosphorylation and altered E2F1-related DNA repair programs.

### 3.8. Inorganic Phosphate Treatment Is Associated with Increased Cisplatin-Induced DNA Damage Accumulation in MDA-MB-231 Cells

To determine whether the Pi-associated attenuation of DNA repair-related responses is accompanied by altered DNA damage levels, a comet assay was performed in MDA-MB-231 cells following 48 h of treatment with NC, Pi, cisplatin, or Pi plus cisplatin.

**Figure 8:**
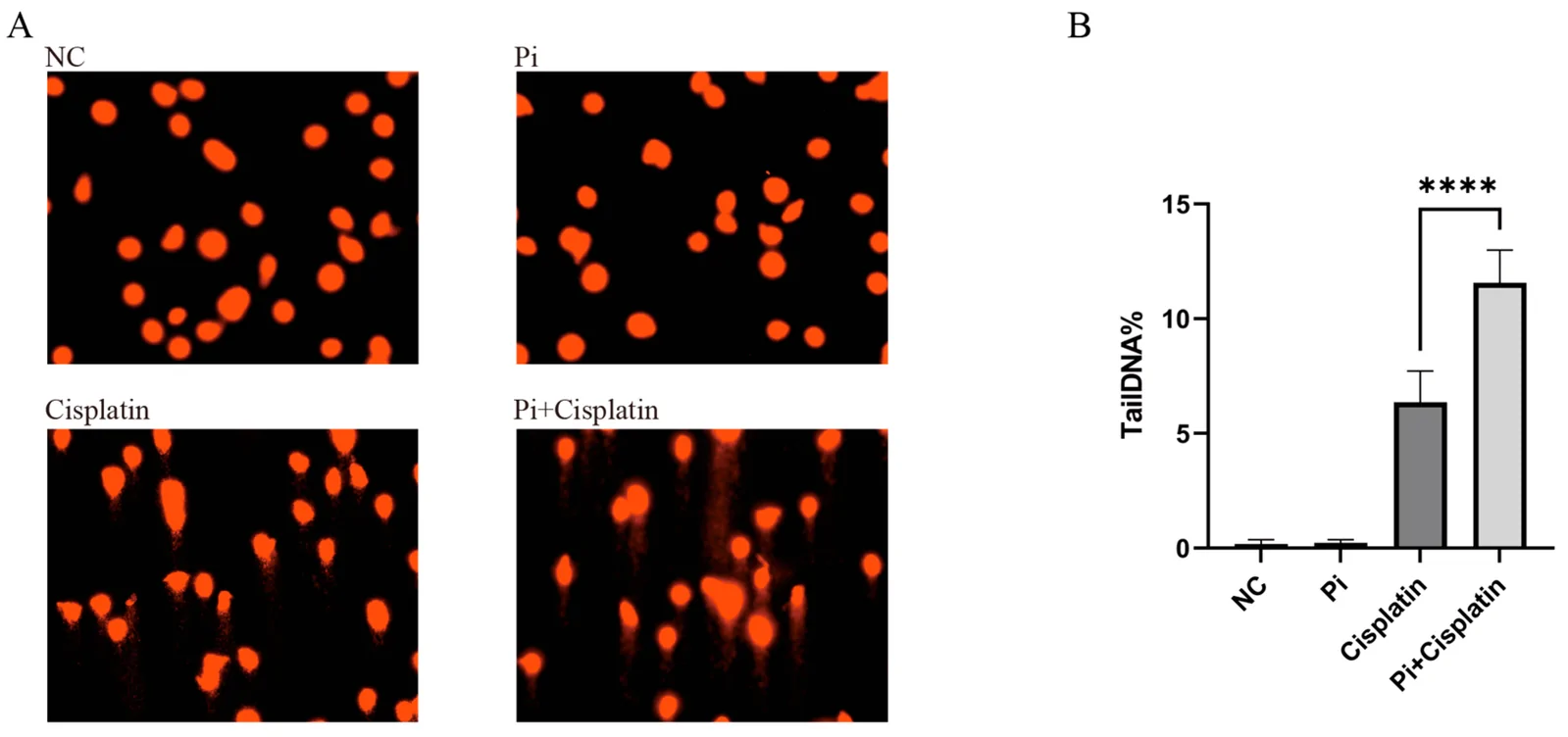
Inorganic phosphate is associated with increased DNA damage accumulation under cisplatin treatment in MDA-MB-231 cells. MDA-MB-231 cells were treated with control (NC), inorganic phosphate (Pi, 5 mM), cisplatin (1 μm ), or a combination of Pi (5 mM) and cisplatin (1 μm ), for 48 h. DNA damage was assessed using an alkaline comet assay. Representative fluorescence images of comet formation are shown (**A**). Quantitative analysis of DNA damage parameters, including tail DNA percentage, was performed based on comet images (**B**). Statistical significance was determined using one-way ANOVA followed by multiple-comparison tests. **** *p* < 0.0001.

As expected, cisplatin treatment alone induced evident DNA damage, as indicated by increased comet tail formation compared with the NC group. In contrast, Pi treatment alone did not result in a significant increase in comet parameters, suggesting that Pi does not markedly induce DNA damage under basal conditions. Notably, combined Pi and cisplatin treatment led to a further increase in comet tail signals compared with cisplatin treatment alone, as reflected by elevated tail DNA percentage, tail length, and olive tail moment. These results indicate that Pi plus cisplatin treatment was associated with increased DNA damage accumulation compared with cisplatin treatment alone in MDA-MB-231 cells (Figure 8A,B).

Taken together with the observed reduction in RB1 phosphorylation and decreased expression of PCNA, RAD51, and multiple DNA repair-related genes, these findings suggest that Pi is associated with increased DNA damage levels. These findings suggest that Pi treatment was associated with reduced DNA repair-related transcriptional and protein-level responses, together with increased DNA damage accumulation under cisplatin treatment. However, further pathway-specific functional assays are needed to determine whether Pi directly affects the activity or capacity of specific DNA repair pathways, such as homologous recombination, nucleotide excision repair, base excision repair, or mismatch repair.

### 3.9. TCGA DNA Repair Pathway Activity and Prognosis

**Figure 9:**
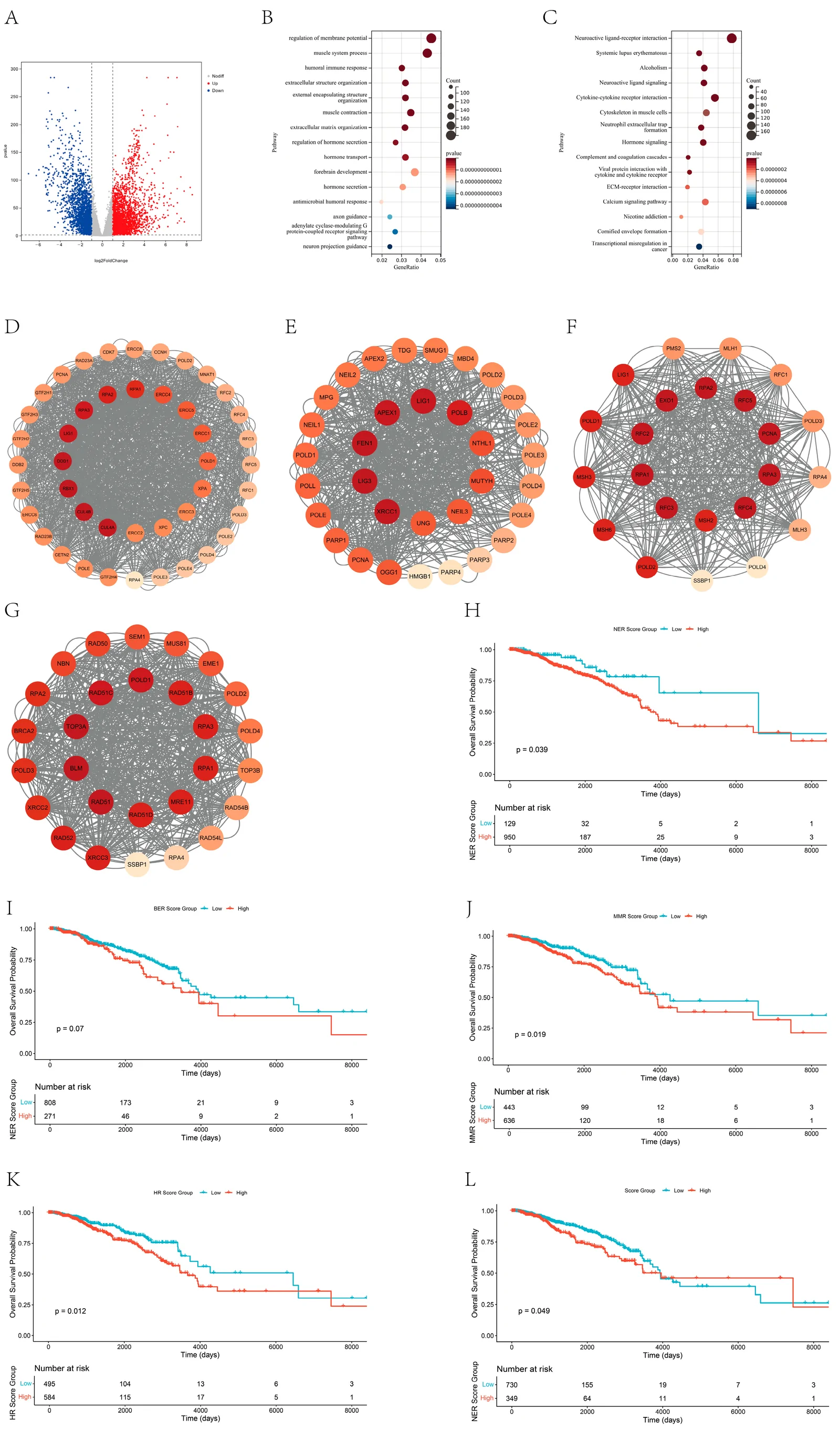
Elevated DNA repair-related gene expression is associated with poor prognosis in breast cancer patients from the TCGA cohort. (**A**) Volcano plot showing differentially expressed genes (DEGs) between breast cancer tissues (*n* = 1077) and adjacent normal tissues (*n* = 99) from the TCGA database. Red and blue dots represent significantly upregulated and downregulated genes, respectively, using thresholds of |log_2_ fold change| > 1 and *p* < 0.05. The horizontal dashed line indicates the significance threshold of p = 0.05 (−log10[p] ≈ 1.30), and the vertical dashed lines indicate the fold-change thresholds of log2FC = −1 and log2FC = 1. (**B**,**C**) Functional enrichment analyses of DEGs, including Gene Ontology (GO) enrichment analysis (**B**) and Kyoto Encyclopedia of Genes and Genomes (KEGG) pathway enrichment analysis (**C**), highlighting biological processes and signaling pathways associated with extracellular structure organization, ion transport, cell migration, immune regulation, and cancer-related signaling. (**D**–**G**) Protein–protein interaction (PPI) networks constructed for four canonical KEGG DNA repair pathways: nucleotide excision repair (NER, (**D**)), base excision repair (BER, (**E**)), mismatch repair (MMR, (**F**)), and homologous recombination (HR, (**G**)). Hub genes were identified based on degree centrality, and the top-ranked genes were selected to represent core repair networks. (**H**–**K**) Kaplan–Meier overall survival (OS) analyses stratifying breast cancer patients into high- and low-expression score groups based on hub-gene–derived repair pathway scores for NER (**H**), BER (**I**), MMR (**J**), and HR (**K**). Repair pathway scores were calculated from the average expression levels of the corresponding hub genes. (**L**) Kaplan–Meier overall survival (OS) analysis based on a custom DNA repair gene set composed of PCNA, RFC1, RFC2, RFC3, POLE, POLD1, and RAD51C. Patients with higher expression scores exhibited significantly worse overall survival.

In this study, transcriptomic data from 1077 breast cancer tumor samples and 99 adjacent normal tissues were obtained from The Cancer Genome Atlas (TCGA) database. Differential expression analysis was performed using DESeq2 with thresholds of |log_2_ fold change| > 1 and *p* < 0.05, identifying a total of 5078 differentially expressed genes (DEGs), including 2983 upregulated and 2095 downregulated genes (Figure 9A).

Gene Ontology (GO) enrichment analysis of the differentially expressed genes (DEGs), ranked by gene count, showed that these genes were primarily enriched in biological processes related to regulation of membrane potential, extracellular structure organization, cell motility-associated processes, humoral immune response, and developmental regulation (Figure 9B).

KEGG pathway analysis showed that the DEGs were mainly enriched in pathways related to receptor-mediated signaling, immune and inflammatory regulation, extracellular matrix interaction, calcium signaling, and cancer-associated transcriptional regulation (Figure 9C). Among these, neuroactive ligand–receptor interaction, hormone signaling, and calcium signaling pathways were significantly enriched, suggesting potential involvement in receptor-mediated signal transduction, secretory regulation, and intracellular calcium homeostasis. Although the global enrichment analysis highlighted multiple biological processes, our subsequent analyses focused on DNA repair-related pathways, given their established roles in genomic stability and their relevance to cisplatin-induced DNA damage observed in our experimental system.

Based on these findings, to further evaluate the clinical relevance of DNA repair pathways in breast cancer, four canonical DNA repair pathways—nucleotide excision repair (NER), base excision repair (BER), mismatch repair (MMR), and homologous recombination (HR)—were selected from the KEGG database. Protein–protein interaction (PPI) networks were constructed for each pathway, and the top 10 genes ranked by degree centrality were identified as core genes, yielding pathway-specific core gene networks (Figure 9D–G).

Kaplan–Meier survival analysis showed that higher pathway activity scores for NER, MMR, and HR were significantly associated with worse overall survival (*p* < 0.05), while BER showed a similar but non-significant trend (Figure 9H–K). In addition, a gene set comprising PCNA, RFC3, RFC1, RFC2, POLE, POLD1, and RAD51C also demonstrated that higher expression levels were associated with poorer overall survival (Figure 9L).

Collectively, these findings indicate that elevated expression of DNA repair-related genes and pathways is associated with unfavorable clinical outcomes in breast cancer. These clinical observations suggest a potential association between DNA repair-related transcriptional activity and tumor aggressiveness. However, the TCGA analysis is based on observational data and does not establish causal relationships.

### 3.10. Inflammation and Cellular ROS

**Figure 10:**
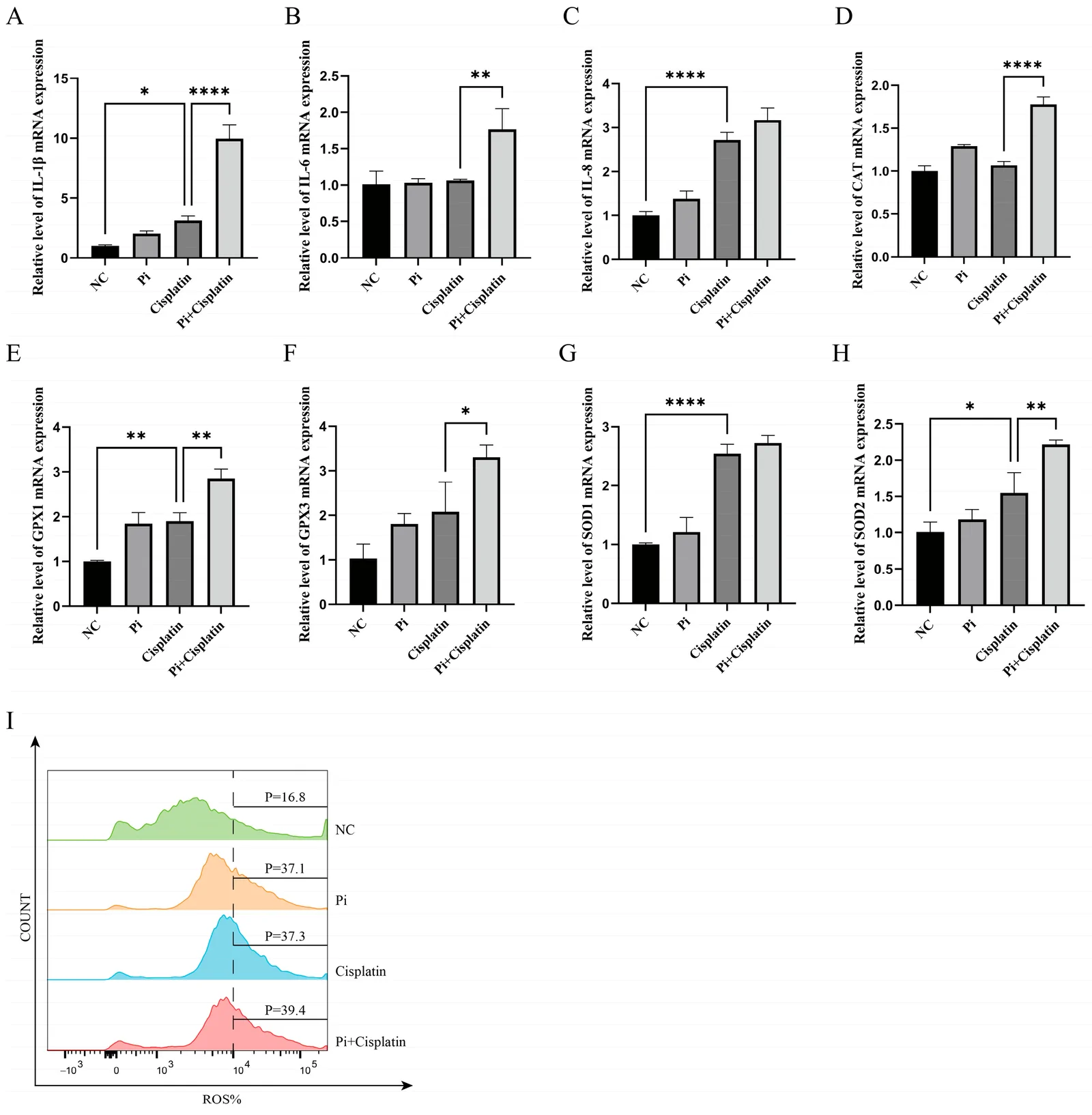
Pi combined with cisplatin is associated with increased inflammatory cytokine expression and intracellular ROS in MDA-MB-231 cells. (**A**–**C**) qPCR analysis of mRNA expression levels of inflammatory cytokines IL-1β, IL-6, and IL-8 in MDA-MB-231 cells treated with negative control (NC), Pi alone, cisplatin alone, or Pi combined with cisplatin for 48 h. Shown are mean ± SD, *n* = 3, one-way ANOVA with multiple comparisons. (**D**–**H**) qPCR analysis of transcripts encoding intracellular and antioxidant ROS-scavenging factors, including CAT, GPX1, GPX3, SOD1, and SOD2, in cells subjected to the indicated treatments for 48 h. Shown are mean ± SD, *n* = 3, one-way ANOVA with multiple comparisons. (**I**) Flow cytometry analysis of intracellular reactive oxygen species (ROS) levels in MDA-MB-231 cells following NC, Pi, cisplatin, or Pi combined with cisplatin treatment for 48 h. Representative histograms and quantitative analysis are shown. * *p* < 0.05, ** *p* < 0.01, **** *p* < 0.0001.

Based on the preceding GO and KEGG enrichment analyses (Figure 3G–I), we further examined the effects of Pi treatment and its combination with cisplatin on inflammatory responses and cellular oxidative stress after 48 h using qPCR and flow cytometry. The expression of inflammatory cytokines was first assessed under different treatment conditions (Figure 10A–C). The results showed that IL-1β and IL-6 expression levels were higher in the Pi + cisplatin group than in the cisplatin-alone group, suggesting an enhanced inflammatory response under combined treatment. In contrast, IL-8 expression was significantly increased in the cisplatin-treated group compared with the NC group, with no significant difference between the Pi + cisplatin and cisplatin-alone groups, indicating that IL-8 induction is primarily associated with cisplatin treatment.

We next evaluated the expression of oxidative stress-related antioxidant genes (Figure 10D–H). The expression levels of CAT, GPX1, GPX3, and SOD2 were higher in the Pi + cisplatin group than in the cisplatin-alone group, suggesting increased oxidative stress-related responses under combined treatment conditions. In contrast, SOD1 expression did not show significant differences among the groups. To further assess oxidative stress, intracellular ROS levels were measured by flow cytometry using DCFH-DA staining (Figure 10I). A rightward shift in ROS signals was observed in the Pi, cisplatin, and Pi + cisplatin groups compared with the NC group, indicating increased intracellular ROS levels in response to both treatments. The combined treatment group did not show attenuation of ROS levels. Collectively, these findings suggest that Pi in combination with cisplatin is associated with increased expression of inflammatory cytokines (IL-1β and IL-6), elevated cellular ROS levels, and upregulation of redox-related genes. These observations are consistent with the enrichment of inflammation- and oxidative stress-related pathways identified in GO and KEGG analyses, providing supportive evidence for the association of these processes under combined treatment conditions.

## 4. Discussion

Triple-negative breast cancer (TNBC) lacks expression of estrogen receptor (ER), progesterone receptor (PR), and human epidermal growth factor receptor 2 (HER2), and therefore does not benefit from endocrine or anti-HER2 targeted therapies [10]. TNBC is characterized by high proliferative activity, aggressive clinical behavior, and a high risk of recurrence, making chemotherapy a central component of treatment [22,23,24]. However, therapeutic responses remain heterogeneous, and acquired resistance frequently limits long-term efficacy [25,26]. Among chemotherapeutic agents, platinum-based drugs such as cisplatin induce DNA crosslinks that disrupt DNA replication and transcription, leading to cell cycle arrest and apoptosis [27,28]. The ability of tumor cells to activate DNA damage response and repair pathways has been implicated in tolerance to platinum-induced cytotoxicity [28,29].

In this study, we observed that inorganic phosphate treatment was accompanied by altered antiproliferative responses under low-dose cisplatin exposure in MDA-MB-231 cells. Multi-level analyses further showed that Pi treatment was associated with G1-phase enrichment, reduced RB1 phosphorylation, lower RB1–E2F module scores, decreased expression of selected DNA repair-associated genes, and increased cisplatin-induced DNA damage accumulation. These findings summarize transcriptional, molecular, and phenotypic changes associated with Pi–cisplatin co-treatment.

Notably, the Pi-associated cellular response was primarily observed under low-dose cisplatin conditions, whereas no additional significant enhancement was detected at higher cisplatin concentrations that already exerted strong antiproliferative effects. Because this study did not systematically evaluate combination effects across a full cisplatin dose matrix, it remains unclear whether the Pi-associated effect is dose-dependent or restricted to specific therapeutic windows. Therefore, although Pi enhanced cisplatin-associated growth inhibition under the specific experimental conditions used in this study, the current data should not be interpreted as demonstrating pharmacological synergy between Pi and cisplatin. Future studies incorporating combination dose–response analyses and Chou–Talalay combination index (CI) calculations will be required to define the effective range, pharmacological interactions, and potential application scenarios of Pi co-treatment.

The RB1–E2F signaling axis is a well-established regulator of G1/S-phase transition and controls the transcription of genes involved in DNA replication and DNA repair [18,30]. In its hypophosphorylated state, RB1 suppresses E2F transcriptional activity, thereby restricting S-phase entry and associated gene expression programs [31,32]. In the present study, combined Pi and cisplatin treatment was associated with reduced RB1 phosphorylation, decreased expression of multiple putative E2F1-associated DNA repair genes, and lower RB1–E2F module scores. These observations are consistent with a model in which Pi-associated changes in cell-cycle progression and DNA repair-related transcription may involve the RB1–E2F axis. However, the module scores represent indirect transcriptomic evidence of altered expression of an RB1–E2F-associated gene program and do not constitute a direct measurement of E2F1 transcriptional activity. Moreover, the present study does not establish a causal requirement for RB1, because RB1 knockdown, knockout, or rescue experiments were not performed. Accordingly, the proposed RB1–E2F–DNA repair relationship should be regarded as a candidate, hypothesis-generating mechanism rather than a definitively established pathway. Future genetic perturbation studies will be required to determine whether RB1 is functionally necessary for the observed phosphate-associated response under cisplatin exposure.

RB1 phosphorylation is primarily regulated by cyclin-dependent kinase complexes, particularly cyclin D–CDK4/6 and cyclin E–CDK2, and may also be influenced by phosphatase activity. Although these upstream regulators were not directly examined, Pi-associated alterations in phosphate sensing, cellular metabolism, or oxidative stress signaling could potentially affect CDK or phosphatase activity and thereby modulate RB1 phosphorylation. Whether phosphate transporters such as PiT1/PiT2, cyclin–CDK complexes, or phosphatases such as PP1 or PP2A mediate this response remains unknown. Future studies combining CDK activity assays, phosphatase analyses, and genetic manipulation of phosphate transporters will be required to identify the upstream events associated with RB1 hypophosphorylation.

It should be noted that the identification of E2F1-associated DNA repair-related genes in this study was based on integrative analysis of publicly available ChIP-seq datasets and pathway annotations. While this approach provides a useful framework for nominating candidate regulatory targets, it does not establish direct E2F1 binding or transcriptional regulation in the specific experimental context of MDA-MB-231 cells under Pi and cisplatin treatment. Therefore, these genes should be considered putative E2F1-associated targets. Direct validation using E2F-responsive luciferase reporter assays, ChIP-qPCR, promoter-reporter assays, or genetic perturbation approaches will be required to confirm these regulatory relationships in future studies.

Transcriptomic and pathway analyses consistently indicated that Pi treatment was associated with reduced enrichment of multiple DNA repair-related gene sets, including mismatch repair, base excision repair, nucleotide excision repair, and homologous recombination pathways. In contrast, cisplatin treatment alone showed a tendency toward increased enrichment of these pathways, consistent with activation of DNA damage response programs. Notably, under combined Pi and cisplatin treatment, DNA repair-related pathway enrichment remained lower than that observed with cisplatin alone. These findings suggest that Pi may be associated with changes in the transcriptional response of DNA repair-associated genes under DNA damage conditions. Consistent with this observation, comet assay results demonstrated increased DNA damage accumulation in the combination group compared with cisplatin treatment alone. However, it is important to emphasize that comet assay reflects net DNA damage levels and does not distinguish between increased damage induction and reduced repair capacity. Future studies using γH2AX foci resolution kinetics or RAD51 foci formation assays, together with pathway-specific DNA repair assays, will be required to determine whether Pi directly affects DNA repair capacity. Therefore, the current findings should be interpreted as evidence of altered DNA damage accumulation and repair-associated responses rather than direct confirmation of impaired homologous recombination activity. Unlike previous studies in which combination strategies enhanced cisplatin cytotoxicity by modulating DNA repair pathway choice—such as suppression of homologous recombination accompanied by compensatory activation of non-homologous end joining—the present study suggests that Pi-associated cellular changes under cisplatin exposure may reflect a distinct transcriptional state, characterized by transcriptional attenuation of multiple DNA repair-associated programs.

To further explore the clinical relevance of DNA repair-related processes, we analyzed TCGA breast cancer datasets and observed that higher expression of DNA repair-related gene sets and pathway scores was associated with poorer overall survival. Similar trends were observed for a core gene set including PCNA, RFC family members, POLE, POLD1, and RAD51C. These findings suggest that elevated DNA repair-related gene expression or pathway scores may be associated with unfavorable clinical outcomes. However, it should be noted that TCGA-BRCA is not a cisplatin-treated cohort and includes heterogeneous breast cancer subtypes. Therefore, these results should be interpreted as exploratory prognostic associations rather than evidence of platinum response prediction. In addition, survival analyses were based on Kaplan–Meier stratification without adjustment for potential confounding clinical variables such as age, tumor stage, and molecular subtype. Consequently, these findings should be considered hypothesis-generating. Validation in independent cohorts, such as METABRIC or other external datasets, together with multivariate Cox regression analyses, will be required in future studies to assess the robustness, generalizability, and independent prognostic value of these DNA repair-related signatures.

In the retrospective clinical cohort, serum inorganic phosphate status was associated with pathological grade. After adjustment for age, TNBC status, and abnormalities in hemoglobin and bicarbonate, abnormal phosphate status remained inversely associated with high-grade tumors, while TNBC status was independently associated with an increased likelihood of grade III disease. Subtype-stratified analyses further showed that abnormal phosphate status was numerically more prevalent in TNBC than in Luminal A, Luminal B, or HER2-positive tumors. Within the TNBC subgroup, the proportion of phosphate abnormality decreased with increasing pathological grade, and this inverse association remained significant in subgroup logistic regression. These findings provide additional clinical context for investigating phosphate-associated cellular responses in a TNBC model. However, they should not be interpreted as evidence that phosphate-associated responses are specific to TNBC or that the observed clinical associations are causal.

Several limitations of the clinical analysis should be acknowledged. First, serum phosphate status was classified as normal or abnormal, with the abnormal category including both hyperphosphatemia and hypophosphatemia. Consequently, the present analysis cannot distinguish whether elevated and reduced phosphate levels exert different biological effects. Second, although age and molecular subtype were incorporated into the multivariable model, other potential confounding factors, including renal function, nutritional status, endocrine regulation, medication exposure, and additional metabolic conditions that may influence serum phosphate homeostasis, were not fully accounted for. Third, detailed treatment information was unavailable, and the retrospective cohort was not designed to evaluate platinum sensitivity or treatment response. Therefore, the current clinical findings should be regarded as associative and hypothesis-generating rather than establishing causal or predictive relationships.

Future studies using well-annotated clinical cohorts with detailed treatment information and longitudinal follow-up will be required to validate these observations. In particular, analyses focusing on TNBC patients receiving platinum-based chemotherapy may help determine whether serum phosphate status is associated with therapeutic response, pathological progression, or clinical outcome, thereby improving the translational relevance of the present findings.

Subtype-specific and treatment-stratified analyses will be performed to determine whether phosphate-associated biochemical features are linked to differential therapeutic sensitivity or disease progression. Furthermore, prospective or longitudinal data collection will be considered to better characterize the temporal relationship between phosphate levels and treatment exposure. Collectively, these efforts will enable a more precise and clinically meaningful assessment of phosphate-associated features in breast cancer and will help determine whether serum phosphate levels may serve as a potential biomarker for predicting treatment response or clinical outcome. In addition to DNA repair-related processes, transcriptomic analyses indicated enrichment of inflammation- and stress-related pathways under combined Pi and cisplatin treatment. Experimental validation showed increased expression of IL-1β and IL-6, along with elevated intracellular ROS levels in the combination group.

Previous studies have shown that elevated Pi levels can induce oxidative stress-related responses in breast cancer cells. For example, high concentrations of Pi have been reported to promote H_2_O_2_ production, alter mitochondrial membrane potential, and activate PKC/NF-κB signaling pathways in MDA-MB-231 cells, whereas these effects are less pronounced in MCF-7 and MCF-10A cells [21,33]. In addition, reactive oxygen species (ROS) are known to play an important regulatory role in cisplatin-induced cytotoxicity. In multiple tumor models, mitochondrial ROS levels have been closely associated with cisplatin-induced apoptosis, with ROS scavenging attenuating cytotoxic effects and ROS elevation enhancing cell death [34]. Beyond nuclear DNA damage, cisplatin can also induce mitochondrial ROS generation, further contributing to its cytotoxic activity [35,36]. In breast cancer models, cisplatin treatment is frequently accompanied by increased ROS levels, mitochondrial membrane depolarization, and enhanced DNA damage [36]. Moreover, elevated inorganic phosphate has been reported to modulate cellular stress responses through mitochondrial ROS production or activation of NADPH oxidase-related pathways [21,37].

These findings provide biological context for the stress-related changes observed in the present study and support the possibility that Pi may be associated with changes in the intracellular stress environment under cisplatin exposure. However, the current data do not establish a causal role for inflammation or oxidative stress in mediating the Pi-associated effect. Moreover, whether ROS functions upstream of RB1–E2F signaling, downstream of RB1–E2F alteration, or represents a parallel stress response remains unresolved in the present study. No perturbation experiments using antioxidants or inflammatory pathway inhibitors were performed. Therefore, these changes are more appropriately interpreted as associated phenotypes rather than confirmed mechanistic drivers. Future studies using agents such as N-acetylcysteine or MitoTEMPO will be required to determine whether oxidative stress contributes functionally to the cellular responses observed under Pi and cisplatin co-treatment.

It should also be emphasized that the extracellular phosphate concentration used in this study (5 mM) exceeds the physiological serum phosphate concentration observed in humans. Therefore, the current experimental conditions were designed to investigate phosphate-associated cellular mechanisms rather than to mimic clinically achievable systemic phosphate levels. Accordingly, the present findings should be interpreted as exploratory observations obtained under elevated extracellular phosphate conditions, and future studies will be required to determine whether similar effects occur at clinically relevant phosphate concentrations or through modulation of phosphate transport and intracellular phosphate signaling.

It is also important to consider that inorganic phosphate is not a biologically inert molecule but plays central roles in cellular metabolism, ATP synthesis, intracellular buffering systems, and phosphorylation-dependent signaling pathways [20,38,39]. Elevated extracellular Pi may modulate cellular processes through multiple mechanisms, including transport via Na^+^-dependent (SLC34, SLC20) and H^+^-dependent phosphate transporters, regulation of metabolic processes and ATP-related dynamics, and induction of reactive oxygen species [40,41,42]. Recent studies further suggest that phosphate transporters, including PiT1/PiT2 (SLC20 family members), may function not only as transporters but also as extracellular phosphate sensors capable of modulating intracellular signaling responses [20,43,44]. In addition, phosphate availability is closely linked to mitochondrial metabolism, ATP production, and redox homeostasis, raising the possibility that elevated extracellular Pi may alter the intracellular metabolic state under cisplatin exposure. Potential physicochemical interactions between Pi and cisplatin, including effects on cisplatin hydrolysis kinetics, ligand exchange reactions, or intracellular distribution, also cannot be excluded. These possibilities were not directly examined in the current study and warrant further investigation.

This study has several limitations. First, functional validation was primarily performed in vitro, and in vivo models are required to assess the effects of Pi on tumor growth and cisplatin response in a physiological context. Second, mechanistic conclusions are largely based on transcriptomic and integrative analyses, and functional validation of key regulatory nodes remains limited. Third, although MCF-7 and MCF-10A cells were included as comparative models in the real-time proliferation assay, all transcriptomic and mechanistic experiments were performed in a single TNBC cell line, MDA-MB-231. Because TNBC is molecularly heterogeneous, these data cannot establish that the observed RB1–E2F- and DNA repair-associated changes are shared across TNBC subtypes. Furthermore, in vitro responses may not fully recapitulate the influence of tumor microenvironmental factors, systemic phosphate regulation, and pharmacokinetic interactions that occur in vivo. The current conclusions have therefore been restricted to the MDA-MB-231 model, and validation in additional TNBC cell lines, patient-derived models, and in vivo cisplatin-response models remains necessary.

Taken together, this study shows that Pi treatment was accompanied by altered antiproliferative responses under cisplatin exposure in MDA-MB-231 cells and was associated with reduced RB1 phosphorylation, lower RB1–E2F module scores, altered expression of DNA repair-associated genes, and increased DNA damage accumulation. Although these observations are consistent with a model linking phosphate-associated cellular changes to cell-cycle regulation and DNA damage-response programs, the underlying causal mechanisms remain to be established. Further studies are required to determine the functional contributions of RB1–E2F signaling, DNA repair capacity, and oxidative stress, as well as the generalizability of these findings across additional TNBC models and in vivo systems. Accordingly, the proposed Pi–RB1–E2F–DNA repair relationship should be interpreted as a model-specific, hypothesis-generating framework based on associated molecular and phenotypic changes rather than as a causally validated signaling cascade.

## 5. Conclusions

**Figure 11:**
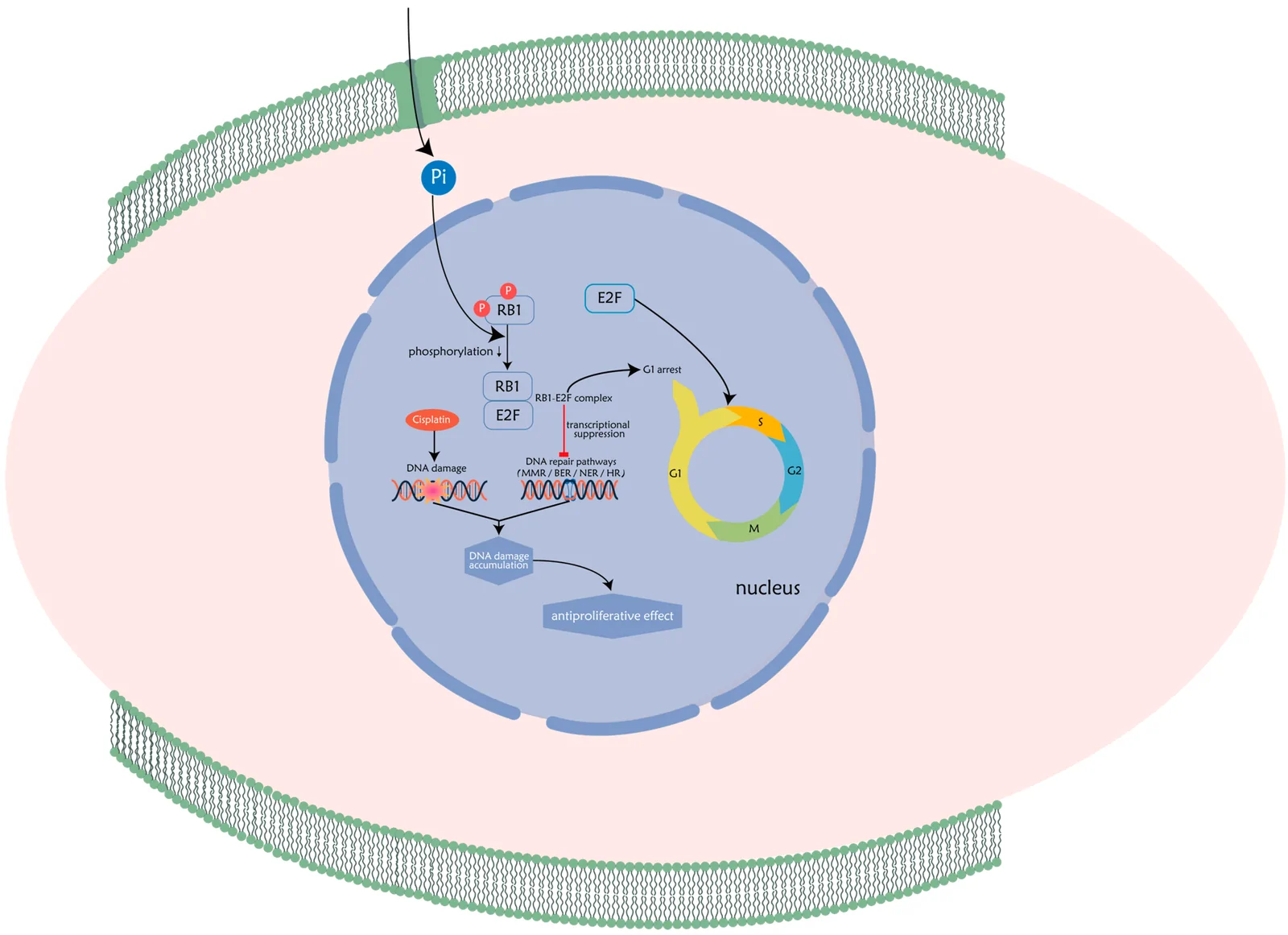
Proposed hypothesis-generating model of phosphate-associated cellular changes under cisplatin exposure in the MDA-MB-231 TNBC model. Elevated extracellular Pi exposure was associated with reduced RB1 phosphorylation. Hypophosphorylated RB1 is generally known to restrain E2F-dependent transcription; however, E2F transcriptional activity was not directly measured in the present study. Instead, lower RB1–E2F module scores and reduced expression of multiple putative E2F1-associated DNA repair genes were observed under Pi-containing treatment conditions. These transcriptional patterns involved genes related to mismatch repair, base excision repair, nucleotide excision repair, and homologous recombination and were accompanied by G1-phase enrichment, increased DNA damage accumulation under cisplatin exposure, and reduced cell proliferation. Collectively, these observations are consistent with a model in which phosphate-associated cellular responses may involve RB1–E2F-associated transcriptional programs and DNA repair-related gene expression. All proposed relationships are based on associative observations and require direct functional validation.

In summary, this study characterizes phosphate-associated cellular responses under cisplatin exposure, primarily in the MDA-MB-231 TNBC model. In a retrospective clinical cohort, serum inorganic phosphate status was associated with pathological grade, indicating a potential relationship between phosphate-related biochemical status and tumor characteristics. Under the experimental extracellular phosphate conditions used in this study, Pi treatment was accompanied by altered proliferation under low-dose cisplatin exposure in MDA-MB-231 cells. At the molecular level, Pi treatment was associated with reduced RB1 phosphorylation, lower RB1–E2F module scores, G1-phase enrichment, reduced enrichment of DNA repair-related gene sets, and increased DNA damage accumulation under cisplatin exposure (Figure 11). In addition, TCGA-based analyses indicated that higher DNA repair pathway scores were associated with poorer overall survival in breast cancer. Collectively, these findings are consistent with a hypothesis-generating model in which phosphate-associated cellular responses may involve RB1–E2F-associated transcriptional programs and DNA repair-related gene expression under cisplatin exposure. The subtype-stratified clinical analyses provide additional clinical context for investigating phosphate-associated responses in TNBC but do not establish TNBC specificity. Accordingly, the proposed Pi–RB1–E2F–DNA repair relationship should be interpreted as a model-specific, hypothesis-generating framework derived primarily from the MDA-MB-231 model rather than as a fully validated signaling cascade. Further studies using additional TNBC models and genetic perturbation approaches will be required to establish its generalizability and mechanistic validity, while independent clinical cohorts will be needed to assess its clinical relevance.

## Data Availability

The RNA sequencing data generated in this study have been deposited in the Gene Expression Omnibus (GEO) database under accession number GSE319411 and will be publicly available upon publication of this article. The breast cancer transcriptomic data analyzed in this study were obtained from The Cancer Genome Atlas (TCGA) project through the Genomic Data Commons (GDC) Data Portal (https://portal.gdc.cancer.gov/).

## Supplementary Materials

The following supporting information can be downloaded at https://www.mdpi.com/article/10.3390/cimb48080839/s1.
