# Supplementary material for: Inorganic Phosphate Is Associated with RB1–E2F-Related Transcriptional and DNA Repair-Associated Changes Under Cisplatin Exposure in MDA-MB-231 Cells
Source: Curr Issues Mol Biol. 2026 Aug 18;48(8):839. doi: 10.3390/cimb48080839 (PMC13511488; doi:10.3390/cimb48080839)
Supplement: Supplementary file 1 [file cimb-48-00839-s001.zip › Supplementary Table S1.pdf]

Table S1. Primer sequences used for RT-qPCR analysis.

RNA	Primer	Sequence
IL-1β	Forward Primer	CCAATCTTCATTGCTCAAGTGTCT
	Reverse Primer	CCATCATTTCACTGGCGAGC
IL-6	Forward Primer	CCTGAACCTTCCAAAGATGGC
	Reverse Primer	CCTGAACCTTCCAAAGATGGC
IL-8	Forward Primer	ATGACTTCCAAGCTGGCCGTGGCT
	Reverse Primer	TCTCAGCCCTCTTCAAAAACTTCTC
CAT	Forward Primer	ATGCAGGACAATCAGGGTGG
	Reverse Primer	AGATCCGGACTGCACAAAGG
GPX1	Forward Primer	AGTCGGTGTATGCCTTCTCG
	Reverse Primer	TCTTGGCGTTCTCCTGATGC
GPX3	Forward Primer	ATGGGCAATCCCCAGATGGAC
	Reverse Primer	GGGAAAGCCCAGAATGACCA
SOD1	Forward Primer	CCTAGCGAGTTATGGCGACG
	Reverse Primer	AGTCTCCAACATGCCTCTCTTC
SOD2	Forward Primer	GCTGGAAGCCATCAAACGTG
	Reverse Primer	GCAGTGGAATAAGGCCTGTTG
RAD51	Forward Primer	AGACCGAGCCCTAAGGAGAG
	Reverse Primer	TCTGCATTGCCATTAGCTCCA
POLD1	Forward Primer	CTGTTTGAAGCGGCAGGATGG
	Reverse Primer	CGAAGCCAGCGAGGATCTATG

POLD3	Forward Primer	CTGAAGAAGTTCGTCCAGAGTG
	Reverse Primer	ACCGATGAATCCACCAGAATTG
PCNA	Forward Primer	TCTTCCCTTACGCAAGTCTCAG
	Reverse Primer	TCATTGCCGGCGCATTTTAG
RFC1	Forward Primer	CCAAGAGTGATAAGGCCGCA
	Reverse Primer	TTACTCCGGGTGTCACTTGC
RFC2	Forward Primer	TGAGGTCCTTCTGGTGGTTTG
	Reverse Primer	AACGTCAATGCCCCTGTCA
RFC3	Forward Primer	GCAAACTCCACAAAGGCTCC
	Reverse Primer	ATTTGGCCACAAACGCTTCC
POLE	Forward Primer	CACCATCCACACCCAGGTCTTC
	Reverse Primer	GTGGTCTGGTCACTGGAAGC
β-actin	Forward Primer	AGAGCTACGAGCTGCCTGAC
	Reverse Primer	AGCACTGTGTTGGCGTACAG
NOX5	Forward Primer	GAAGGCTGTAGAGGCACCAT
	Reverse Primer	TGAGTTTGTCCATGGGGCTG
